# Molecular characterization of gliomas and glioneuronal tumors amid Noonan syndrome: cancer predisposition examined

**DOI:** 10.3389/fonc.2024.1453309

**Published:** 2024-09-06

**Authors:** Margaret Shatara, Kathleen M. Schieffer, Marilena Melas, Elizabeth A. Varga, Diana Thomas, Brianna A. Bucknor, Heather M. Costello, Gregory Wheeler, Benjamin J. Kelly, Katherine E. Miller, Diana P. Rodriguez, Mariam T. Mathew, Kristy Lee, Erin Crotty, Sarah Leary, Vera A. Paulson, Bonnie Cole, Mohamed S. Abdelbaki, Jonathan L. Finlay, Margot A. Lazow, Ralph Salloum, Maryam Fouladi, Daniel R. Boué, Elaine R. Mardis, Catherine E. Cottrell

**Affiliations:** ^1^ The Division of Hematology and Oncology, St. Louis Children’s Hospital, Washington University School of Medicine in St. Louis, St. Louis, MO, United States; ^2^ The Steve and Cindy Rasmussen Institute for Genomic Medicine, Nationwide Children’s Hospital, Columbus, OH, United States; ^3^ Department of Pathology, The Ohio State University, Columbus, OH, United States; ^4^ Department of Pediatrics, The Ohio State University, Columbus, OH, United States; ^5^ Department of Pathology and Laboratory Medicine, Nationwide Children’s Hospital, Columbus, OH, United States; ^6^ The Department of Radiology, Nationwide Children’s Hospital, Columbus, OH, United States; ^7^ Division of Pediatric Hematology, Oncology, Bone Marrow Transplant and Cellular Therapy, Department of Pediatrics, Seattle Children’s Hospital, University of Washington, Seattle, WA, United States; ^8^ Department of Laboratory Medicine and Pathology, University of Washington School of Medicine, Seattle, WA, United States; ^9^ The Division of Hematology/Oncology, and Bone Marrow Transplantation, Nationwide Children’s Hospital and The Ohio State University, Columbus, OH, United States

**Keywords:** glioma, glioneuronal tumor, Noonan syndrome, cancer predisposition, PTPN11, germline, genomic profiling, next-generation sequencing

## Abstract

**Introduction:**

In the setting of pediatric and adolescent young adult cancer, increased access to genomic profiling has enhanced the detection of genetic variation associated with cancer predisposition, including germline syndromic conditions. Noonan syndrome (NS) is associated with the germline RAS pathway activating alterations and increased risk of cancer. Herein, we describe our comprehensive molecular profiling approach, the association of NS with glioma and glioneuronal tumors, and the clinical and histopathologic characteristics associated with the disease.

**Methods:**

Within an institutional pediatric cancer cohort (n = 314), molecular profiling comprised of paired somatic disease–germline comparator exome analysis, RNA sequencing, and tumor classification by DNA methylation analysis was performed.

**Results:**

Through the implementation of paired analysis, this study identified 4 of 314 (1.3%) individuals who harbored a germline *PTPN11* variant associated with NS, of which 3 individuals were diagnosed with a glioma or glioneuronal tumor. Furthermore, we extend this study through collaboration with a peer institution to identify two additional individuals with NS and a glioma or glioneuronal tumor. Notably, in three of five (60%) individuals, paired genomic profiling led to a previously unrecognized diagnosis of Noonan syndrome despite an average age of cancer diagnosis of 16.8 years. The study of the disease-involved tissue identified signaling pathway dysregulation through somatic alteration of genes involved in cellular proliferation, survival, and differentiation.

**Discussion:**

Comparative pathologic findings are presented to enable an in-depth examination of disease characteristics. This comprehensive analysis highlights the association of gliomas and glioneuronal tumors with RASopathies and the potential therapeutic challenges and importantly demonstrates the utility of genomic profiling for the identification of germline cancer predisposition.

## Introduction

Noonan syndrome (NS) is an autosomal dominant multisystem disorder with an estimated incidence of 1:1,000 to 1:2,500 in which affected individuals display characteristic facial features, congenital heart defects, short stature, developmental delays, and an increased risk of malignancy (1). NS is caused by pathogenic variation in the RAS/MAPK signaling pathway, which leads to aberrant pathway activation (2). More than 50% of individuals with NS have a germline pathogenic variant in *PTPN11* (3, 4). Other genes in the RAS/MAPK signaling pathway (i.e., *A2ML1*, *BRAF*, *CBL*, *HRAS*, *KRAS*, *LZTR1*, *MAP2K1*, *MAP2K2*, *NRAS*, *RASA2*, *RIT1*, *RAF1*, *SHOC*, *SOS1*) are also implicated in NS or related constitutional disease with overlapping phenotypes, collectively termed RASopathies (2, 3). Leukemias, solid tumors, and central nervous system (CNS) tumors are reported with increased frequency in individuals diagnosed with a RASopathy (5–9). Among CNS tumors, gliomas and glioneuronal tumors are reported in association with NS, including but not limited to dysembryoplastic neuroepithelial tumor (DNET), rosette-forming glioneuronal tumor (RGNT), and pilocytic astrocytoma. Notably, molecular analysis has identified concurrent somatic activation of the RAS/MAPK and PI3K/AKT signaling pathways in gliomas, which intriguingly overlap the signaling dysregulation seen in RASopathies (10–12).

The World Health Organization (WHO) 2021 guidelines (13) have expanded the defining molecular features associated with gliomas and, in concert with histopathologic findings, can provide additional supporting data for tumor classification in the setting of an integrated diagnosis. For example, RGNT is a rare, indolent, glioneuronal WHO grade 1 tumor (13), characterized by biphasic neurocytic and glial architecture, most commonly arising in the midline, especially in the fourth ventricular region (14–16), with rare cases reported in the spine or cerebral hemispheres (17, 18). Molecularly, RGNT shares a characteristic methylation signature and *FGFR1* alterations, co-occurring with alterations involving *PIK3CA* or *PIK3R1* and/or *NF1* (12, 19, 20). Diffuse leptomeningeal glioneuronal tumor (DLGNT) is an example of a glioma that was introduced as a provisional entity in the 2016 WHO Classification of CNS Tumors (21). DLGNT is a rare glioneuronal neoplasm, composed of oligodendrocyte-like cells and molecularly characterized by chromosome arm 1p deletion with MAPK pathway gene alteration (13).

Historically, treatment for pediatric low-grade gliomas (LGGs) included surgical resection, radiation therapy, and adjuvant chemotherapy (22–26). Unlike their adult counterparts, pediatric LGGs tend to have a favorable prognosis, with rare reports of malignant transformation (26, 27). Pediatric LGGs typically harbor alterations that result in RAS/MAPK signaling pathway activation for which targeted inhibitors, such as the combination of dabrafenib (BRAF inhibitor) and trametinib (MEK inhibitor), have demonstrated utility as a first-line treatment (11, 28, 29). High-grade gliomas (HGGs) harbor alterations affecting various signaling pathways (30). Clinical trials are underway to determine the efficacy of various targeted therapies against pediatric HGGs (31).

Herein, we present a series of five individuals with NS diagnosed with glioma or glioneuronal tumors, along with molecular characterization of these tumors. The integrated diagnosis presented draws from the clinicopathologic, histologic, and molecular features of the tumors from these individuals.

## Materials and methods

### Cohorts

At the Nationwide Children’s Hospital (NCH; Columbus, OH), 314 individuals with solid tumors or hematologic malignancy/disease consented as part of an institutional translational protocol from January 2018 to April 2022. Under this Institutional Review Board (IRB)-approved protocol (IRB17-00206), comprehensive molecular profiling, including paired exome sequencing of tumor tissue and a germline comparator, RNA sequencing of the tumor, and when possible, DNA methylation array-based tumor classification, was performed. A histopathologic review of a hematoxylin and eosin-stained slide was used to estimate tumor content among the assayed tumor specimens. This protocol allowed for the identification of somatic cancer-associated genomic alterations to include single nucleotide variants (SNVs) and small insertion-deletion (indel) events, copy number alterations, gene fusions, and importantly, germline findings associated with cancer predisposition. Individuals 1–3 were profiled as part of the NCH cohort methodology.

To expand the results and analysis of the NCH cohort, the Seattle Children’s Hospital (Seattle, WA) contributed data from two adolescent young adult individuals who underwent molecular profiling as part of routine clinical testing in the setting of a CNS malignancy at the University of Washington (Seattle, WA). The contribution of this dataset was conducted under an IRB-approved protocol (#14449). Individuals 4 and 5 were profiled as part of the Seattle cohort methodology.

### Enhanced exome sequencing—NCH cohort

Libraries were prepared using 100 ng of input DNA beginning with enzymatic fragmentation, followed by end repair, 5′ phosphorylation, A-tailing, and platform-specific adapter ligation using NEBNext Ultra II FS reagents (New England Biolabs, Ipswich, MA). Target enrichment by hybrid capture was performed using the xGen Exome Research Panel v1.0 enhanced with the xGenCNV Backbone and Cancer-Enriched Panels-Tech Access (Integrated DNA Technologies, Coralville, IA). Paired-end 151-bp reads were generated on the Illumina HiSeq 4000 or NovaSeq 6000 (Illumina, San Diego, CA). Secondary analysis was performed using Churchill, a comprehensive bioinformatic workflow that takes raw sequencing reads from alignment through variant identification through the incorporation of the following analytical steps (32). Reads were aligned to the human genome reference sequence (build GRCh38) using Burrows-Wheeler Aligner (BWA) (v0.7.15) and refined according to community-accepted guidelines for best practices (https://gatk.broadinstitute.org/hc/en-us). Duplicate sequence reads were removed using samblaster-v.0.1.22, and base quality score recalibration was performed on the aligned sequence data using the Genome Analysis Toolkit (v4.1.9) (33). The average depth of coverage obtained for the comparator germline samples and disease-involved tumors is presented in **Supplementary Table 1**. Germline variants were called using GATK’s HaplotypeCaller (34). Somatic SNV and indel detection was performed using MuTect2 (35). Variants were filtered for those >5% variant allele fraction (VAF), within a coding region of the genome, or in proximity to an exon/intron junction (splice site region ≤ 3 bp), with a parameter defined as passing (MuTect2 PASS), and according to population frequency (gnomAD FAFpopmax95 <= 0.0001). Additionally, germline and somatic variation in cancer-associated genes was filtered using a gene set curated from the published literature and genomic databases including those described by Zhang et al., as well as genes with strong or emerging evidence of germline or somatic cancer association as documented in the Cancer Gene Census (36, 37) (**Supplementary Table 2**). Variants flagged as pathogenic/likely pathogenic or with conflicting interpretations in ClinVar were also reviewed. Reportable variants were manually reviewed using the Integrated Genomics Viewer. Variants reported as part of this translational research protocol met the aforementioned criteria and were evaluated in the setting of the tumor type under study. Variants of uncertain significance were not reported. Copy number variation (CNV) was assessed using a combination of GATK (v4.2.4.1) and VarScan2 (38).

### RNA sequencing—NCH cohort

In parallel with enhanced exome sequencing, tumor-derived RNA, input at 500 ng, was subject to DNase treatment and ribodepletion prior to library preparation using the NEBNext Ultra II Directional RNA Library prep kit with 5–10 min of chemical fragmentation for individuals 1, 2, and 3. Independent cDNA libraries were diluted for whole transcriptome sequencing. Paired-end 151-bp reads were generated on the Illumina HiSeq 4000 or NovaSeq 6000 (Illumina, San Diego, CA), and reads were aligned to the human genome reference sequence (GRCh38). The resultant output represented 141,857,623 (individual 1), 236,186,711 (individual 2), and 185,159,431 (individual 3) uniquely mapped reads for the tumor samples.

For fusion analysis, RNA sequence data were processed using EnFusion (39), an ensemble approach of seven fusion callers STARfusion (v.1.6.0) (40), MapSplice (v.2.2.1) (41), FusionCatcher (v.0.99.7c) (42), FusionMap (v.mono-2.10.9) (43), JAFFA (v.1.09) (44), CICERO (v0.3.0) (45), and Arriba (v1.2.0) (46). Rare fusions (<5% frequency in our internal cohort) identified by at least three fusion callers were subject to further review for biological relevance.

Gene expression analyses were performed in R-4.1.1. using the R packages tidyverse (v.1.3.1), dplyr (v.1.0.9), and plyr (v.1.8.7) for data manipulation; Salmon (v.1.9.0), tximport (v.1.18.0), matrixStats (v.0.61.0), umap (v.0.2.8.0), and Rtsne (v.0.16) for analytics; and rsvg (v.2.2.0), ggrepel (v.0.9.1), RColorBrewer (v.1.1.3), plotly (v.4.10.0), and ggplot2 (v.3.3.6) for analytic visualization.

Normalized read counts (TPM) were calculated for all samples from an internal NCH cohort of CNS cancers (*n* = 235) using the Salmon package. An external cohort of CNS cancers (*n* = 791) from the Treehouse Childhood Cancer Initiative at the University of California Santa Cruz Genomics Institute (v.9 and v.11, University of California Santa Cruz, Santa Cruz, CA) was combined with the internal cohort to improve clustering. Expression counts of protein-coding genes were log2(*x* + 1)-transformed and quantile normalization was performed. The 500 protein-coding genes with the highest variances were used to perform a principal components analysis. The data dimensionality was further reduced by performing Uniform Manifold Approximation & Projection (UMAP) using the umap package. This analysis used the first 34 principal components, as they account for >80% of the variance among the samples. The UMAP plot of UMAP1 and UMAP2 was generated using the R packages ggplot2 and plotly.

Scripts used for RNASeq fusion calling and dimensionality reduction can be found at https://github.com/nch-igm/EnFusion and https://github.com/nch-igm/RNAseq_dimensionality_reduction, respectively.

### DNA methylation array—NCH cohort

DNA methylation profiling was performed on nucleic acid extracted from disease-involved tissue. For each tumor studied, 250 ng of input DNA was bisulfite-converted (catalog # D5006, Zymo Research, Irvine CA) and, if applicable, treated using the Illumina formalin-fixed paraffin-embedded (FFPE) restoration process (catalog # WG-321-1002, Illumina, San Diego CA). Bisulfite-converted DNAs, including methylated human DNA controls (catalog # D5014, Zymo Research, Irving CA), were hybridized to the Infinium Methylation EPIC BeadChip (catalog # WG-317-1001, Illumina, San Diego, CA) following the Illumina Infinium HD Methylation protocol. Beadchips were imaged on the Illumina iScan System, and the resulting raw IDAT files were processed through a local installation of the German Cancer Research Center (DKFZ) DNA Methylation Brain Tumor Classifier, version 12.5 (47). The Classifier algorithm produces a score that indicates the similarity of the queried sample against previously characterized CNS tumor types, wherein a score ≥0.9 is strongly supportive of that type.

### Targeted next-generation sequencing—Seattle cohort

Paired tumor–germline testing was performed on disease-involved and normal sample DNA from individuals 4 and 5 using the University of Washington-OncoPlex version 7 (OPXv7), a DNA-based targeted next-generation sequencing panel, as previously described (48). In brief, DNA was extracted from peripheral blood using Qiagen DSP DNA Midi Kit, while total nucleic acid (TNA) was extracted from FFPE tissue using the Qiagen DNA/RNA AllPrep Kit followed by DNA extraction using the Qiagen GeneRead DNA FFPE Kit (Qiagen, Valencia, CA). After shearing, libraries prepared using KAPA HyperPrep reagents (Roche, Wilmington, MA) were hybridized to a set of custom probes (xGen Lockdown Probes, Integrated DNA Technologies, Coralville, IA) designed to target a panel of 377 genes (**Supplementary Table 3**) chosen for their relevance in cancer diagnosis, prognosis, and/or treatment. In addition to identifying SNVs, indels, select gene fusions, and CNVs, OPXv7 was also validated to detect microsatellite instability and tumor mutational burden. The libraries were subsequently sequenced on an Illumina NextSeq500 system (Illumina, San Diego, CA), and sequences were processed through an automated, custom-designed bioinformatics pipeline developed by the University of Washington Next Generation Sequencing Laboratory and Analytics group before analysis by a board-certified molecular pathologist (48). The average depth of coverage obtained for the comparator germline samples and disease-involved tumors is presented in **Supplementary Table 1**.

## Results

Amid the NCH cohort, 4 of 314 individuals (1.3%) who underwent genomic profiling as part of an institutional translational cancer protocol harbored a germline variant in *PTPN11* associated with NS. A diagnosis of NS was not previously known in two of the four (50%) individuals within this cohort until comprehensive molecular profiling was performed due to a cancer diagnosis. Three of these individuals were diagnosed with CNS malignancies including RGNT (*n* = 2; individuals 1 and 2) and a diffuse leptomeningeal glioneuronal tumor with worrisome molecular features, not elsewhere classified (NEC) (*n* = 1; individual 3). A fourth individual with NS in the NCH cohort was diagnosed with B-cell acute lymphocytic leukemia and is therefore excluded from further in-depth examination in this CNS-focused analysis. The paired exome sequencing used in this translational protocol testing was the first manner of identification of NS-associated *PTPN11* variation in individuals 2 and 3 and subsequently allowed for orthogonal confirmation by targeted Sanger sequencing in the clinical laboratory. The observation of CNS malignancies and NS in the NCH cohort served as the foundation for an extended analysis. To further enhance our dataset, collaboration with a peer institution identified two additional individuals diagnosed with gliomas or glioneuronal tumors and NS (Seattle cohort—individuals 4 and 5). Given the unique molecular profiles associated with these CNS tumors, we discuss the utility of paired sequencing and the clinicopathologic, histologic, and molecular features of these five individuals. The average age at cancer diagnosis among this combined cohort of five individuals with NS was 16.8 years (range 9–22 years). The cohort was predominantly male patients (one female patient, four male patients) with variable clinical features associated with NS, including short stature (five of five), dysmorphic facial features (five of five), intellectual disability or delay (four of five), and cardiac abnormalities (three of five) (**Supplementary Table 4**).

### Individual 1

**Figure 1:**
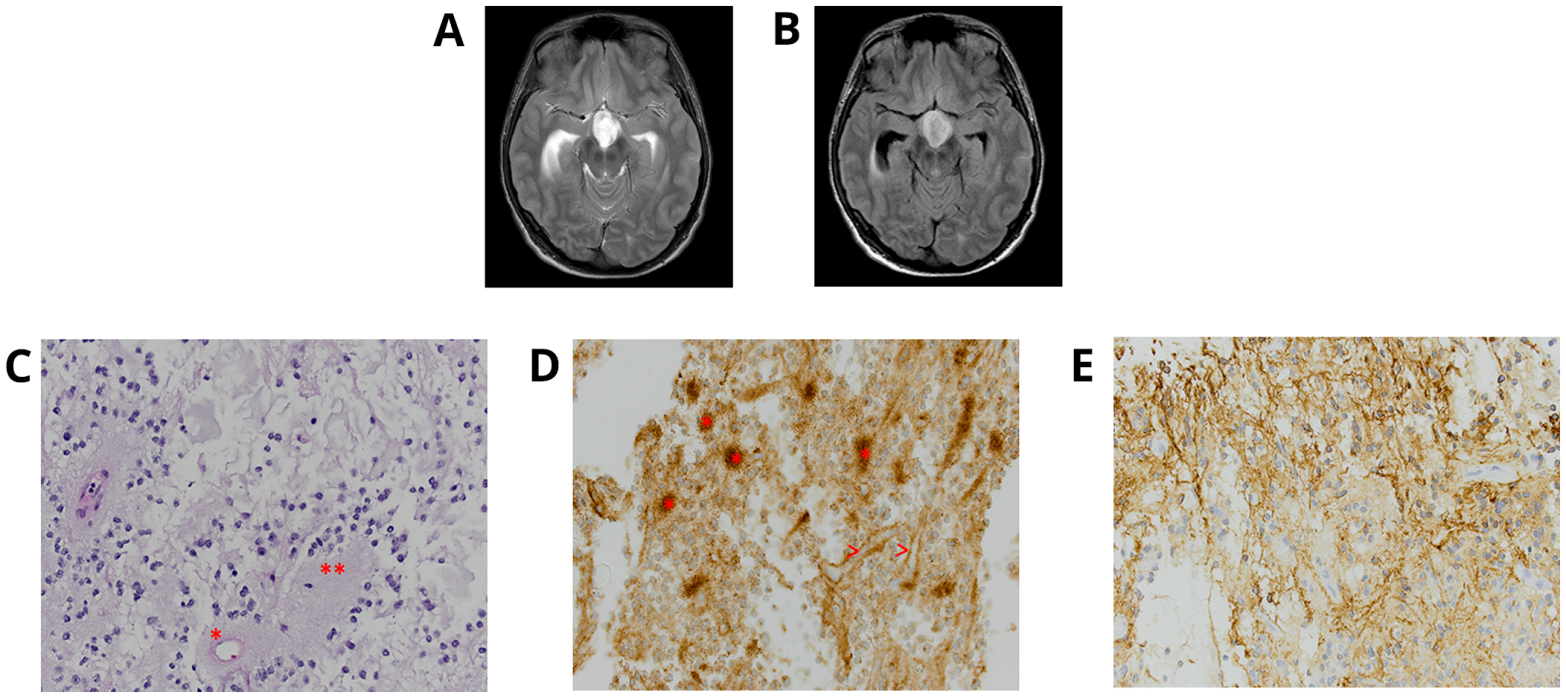
Individual 1—radiologic and histopathologic studies of the tumor. Axial T2 **(A)** and FLAIR **(B)**-weighted MRI brain displays an extensive suprasellar mass involving the third ventricle and extending through the floor into the prepontine cistern with obstructive hydrocephalus. A rosette-forming glioneuronal tumor (RGNT) dominated by oligodendroglia-like, small round neurocytic cells, within a mucinous-myxoid stroma, shows **(C)** neurocytic cell rosettes surrounding an eosinophilic neuropil core (**) and a perivascular neuropil-accumulating pseudo-rosette (*) [H&E ×40 ocular mag.] and **(D)** also demonstrates the synaptophysin-reactivity of many neuropil cores (>) [synaptophysin, ×40 ocular mag.]. **(E)** Area of biphasic tumor with more glial tumor cell component is strongly positive for GFAP [GFAP, ×40 ocular mag.].

A 22-year-old male patient with NS diagnosed during infancy presented with progressive headaches, vomiting, and visual disturbances. A brain magnetic resonance imaging (MRI) demonstrated an extensive suprasellar mass involving the third ventricle, extending through the floor into the prepontine cistern with obstructive hydrocephalus (**Figures 1A, B**). Spinal MRI showed no evidence of drop metastasis. Subtotal surgical resection was performed. Histologic examination of the tumor (**Table 1**) showed a low-grade glioma of moderate cell density, composed of mostly small bland round neurocytic-type cells, with fewer bipolar-spindled piloid cells, consistent with a partial pilocytic astrocytoma component; focally, ganglion-like cells were also seen. There was a partial angiocentric (perivascular rosette-like) pattern, with small round neurocytic cells surrounding perivascular neuropil-rich regions, which showed staining for synaptophysin (**Figures 1C–E**). In some areas, rosettes with eosinophilic neuropil cores surrounded by completely circumferential neurocytic tumor cells were obvious. Occasional eosinophilic granular bodies were seen focally, but no Rosenthal fibers were present. There was prominent glomerular-type microvascular proliferation and focal microcalcification.

**Table 1:** Comparative pathologic findings among the described tumors in this cohort.

Histopathological feature	Individual 1 RGNT	Individual 2 RGNT	Individual 3 Glioneuronal neoplasm with worrisome molecular features, NEC	Individual 4 Low-grade glioneuronal tumor, NEC	Individual 5 DNET
**Rosenthal fibers**	No	No	No	No	No
**Eosinophilic granular bodies**	Yes	No	No	No	No
**Neurocytic cells**	Yes	Yes	No	No	No
**Ganglion cells**	Yes	No	No	No	Yes
**Mitoses**	Few	No	No	No	No
**Microvascular proliferation**	Yes	No	No	Yes	No
**Hyalinized vessels**	No	No	No	No	No
**Microcalcification**	Yes	No	No	Yes	No
**Mucinous microcystic matrix**	Yes	No	No	Yes, focal	Yes
**Tumor infiltration**	None	Focal	Dural and leptomeningeal involvement	None	Yes
**Perivascular pseudorosettes**	Yes	No	No	No	No
**Rosettes with neuropil cores**	Yes	Yes	No	No	No
**Ki-67 index**	5%–10%	<3%	<5%	<3%	<3%
**GFAP**	Partially positive	Partially positive	Focally positive	Positive	Partially positive
**Synaptophysin**	Partially positive	Partially positive	Highlights very rare cells	Partially positive	Partially positive

DNET, dysembryoplastic neuroepithelial tumor; NEC, not elsewhere classified; RGNT, rosette-forming glioneuronal tumor.

Comprehensive genomic profiling using paired exome sequencing confirmed the presence of a germline missense variant in *PTPN11* p.Gly60Ala (classified as pathogenic in ClinVar, variation ID: 40493) (**Table 2**). Additionally, the tumor (estimated at 100% tumor content) was found to harbor an in-frame deletion in *PIK3R1* p.Ile442_Thr454del and a recurrent hotspot missense variant in *FGFR1* p.Lys656Glu. No clinically actionable gene fusions were identified. No CNV or copy-neutral loss of heterozygosity (cnLOH) was identified (**Supplementary Figure 1A**). DNA methylation array-based classification analysis (v12.5) demonstrated a high-confidence family score (0.99907) for a low-grade glioneuronal tumor, with the highest methylation class associated with RGNT (0.56372).

**Table 2:** Clinical and molecular characteristics among this cohort.

Patient (integrated diagnosis)	Sex	Age at Noonan syndrome diagnosis (y)	Age at cancer diagnosis (y)	Germline findings (VAF)	Somatic findings (VAF)	DNA array-based methylation classification (v12.5)
**Individual 1 (RGNT)**	M	5	22	*PTPN11* NM_002834.3:c.179G>C:p.Gly60Ala (50%)	*FGFR1* NM_023110.2:c.1966A>G: p.Lys656Glu (44%) *PIK3R1* NM_181532.2: c.1324_1362delATTGAAGCTGTAGGGAAAAAATTACATGAATATAACACT:p.Ile442_Thr454del (25%)	Rosette-forming glioneuronal tumor (class score: 0.56372)
**Individual 2 (RGNT)**	M	19.5	19	*PTPN11* NM_002834.3:c.922A>G:p.Asn308Asp (46%)	*FGFR1* NM_023110.2:c.1638C>T: p.Asn546Lys (32%) *PIK3R1* NM_181523.2:c.1353_1358delATATAA:p.Glu451_Asn453delinsAsp (34%) *PIK3R1* NM_181523.3: c.1359_1367delCACTCAGTT: p.Thr454_Phe456del (7%) *PIK3R1* NM_181523.2:c.2083G>A: p.Val695Met (43%)	Rosette-forming glioneuronal tumor (class score: 0.99998)
**Individual 3 (glioneuronal neoplasm with worrisome molecular features, NEC)**	F	9	9	*PTPN11* NM_002834.3:c.209A>G: p.Lys70Arg (45%)	*KRAS* NM_004985.5:c.35G>A: p.Gly12Asp (13%) Loss of chromosome arm 1p Gain of chromosome arm 1q 4q12 amplification (*PDGFRA*, *KIT*, *KDR*, *CHIC2*, and *FIP1L1*)	Meningioma, benign, (class score: 0.35455); clinically reported as no match
**Individual 4 (low-grade glioneuronal tumor, NEC)**	M	18.9	18	*PTPN11* NM_002834.3:c.922A>G: p.Asn308Asp (50%)	*FGFR1* NM_023110.2:c.1638C>A: p.Asn546Lys (26%) *PIK3CA* NM_006218.2:c.3140A>G: p.His1047Arg (25%) *PIK3CA* NM_006218.2:c.3139C>T: p.His1047Tyr (5%)	N/A
**Individual 5 (DNET)**	M	1.5	16	*PTPN11* NM_002834.3:c.174C>A: p.Asn58Lys (43%) *EGFR* NM_005228.3:c.1591C>T:p.Arg531Ter (47%)	*FGFR1* NM_023110.2:c.1638C>A p.Asn546Lys (10%)	No match

DNET, dysembryoplastic neuroepithelial tumor; NEC, not elsewhere classified; RGNT, rosette-forming glioneuronal tumor.

The final integrated diagnosis was consistent with RGNT (WHO grade 1). A year following tumor resection, focal proton beam irradiation was performed for local progression, which was complicated by severe somnolence syndrome and managed with steroid therapy. There has been no evidence of tumor progression 38 months after irradiation.

### Individual 2

**Figure 2:**
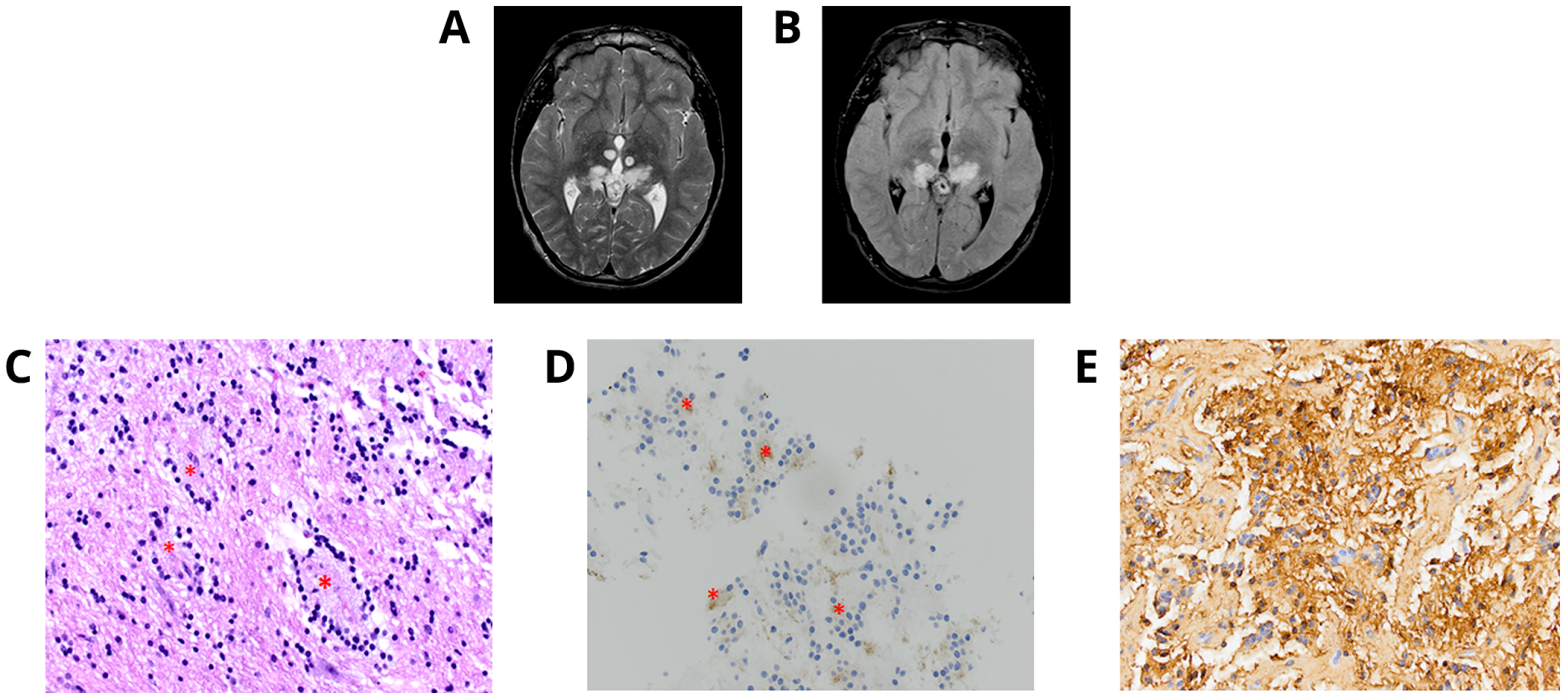
Individual 2—radiologic and histopathologic studies of the tumor. Axial T2 **(A)** and FLAIR **(B)**-weighted MRI brain demonstrates multiple abnormal signals in the pineal region, thalami, and midbrain. **(C)** RGNT with neurocytic cells of moderate density near-completely surrounding neuropil cores (*) [H&E, ×40 ocular mag.]. **(D)** Synaptophysin stain shows neurocytes surrounding synaptophysin-positive neuropil cores (*) [synaptophysin, ×40 ocular mag.]. **(E)** Area of biphasic tumor with more so glial tumor cells and matrix is diffusely positive for GFAP [GFAP, ×40 ocular mag.].

A 19-year-old male patient was reported with complex medical history including prematurity, bradycardia, and cyanosis at birth requiring cardiopulmonary resuscitation, reported neonatal strokes, developmental delays, facial dysmorphism, short stature, and intellectual disability. He presented with chronic migraine headaches, and brain imaging at the age of 16 years displayed multiple abnormal signals in the pineal region, thalami, and midbrain of unknown etiology (**Figures 2A, B**). Spine MRI and cerebrospinal studies were negative. An MRI-guided stereotactic biopsy was consistent with RGNT; bland small round neurocytic cells of moderate density, with no significant pleomorphism, were found completely surrounding synaptophysin-positive neuropil cores, with no Rosenthal fibers or eosinophilic granular bodies seen (**Table 1** and **Figures 2C–E**).

Paired exome sequencing (estimated 70% tumor content) identified a pathogenic missense hotspot variant in *PTPN11* p.Asn308Asp (classified as expert panel pathogenic in ClinVar, variation ID: 13326) (**Table 2**). This variant was subsequently confirmed by targeted Sanger sequencing in our clinical laboratory and reported in the patient’s medical record. Molecular characterization was therefore consistent with NS, a diagnosis which was previously unrecognized in this individual. The somatic analysis revealed three alterations within *PIK3R1*. These include the missense variant, p.Val695Met, an in-frame indel p.Glu451_Asn453delinsAsp, and lastly, an in-frame deletion p.Thr454_Phe456del. The two in-frame indels (p.Glu451_Asn453delinsAsp and p.Thr454_Phe456del) did not occur within the same read based on visualization of the aligned sequence (**Supplementary Figure 2**). Additionally, a recurrent hotspot missense variant in *FGFR1* p.Asn546Lys was also detected. No clinically actionable gene fusions were identified. No CNV was identified; however, cnLOH involving chromosome 19p was detected (**Supplementary Figure 1B**). DNA methylation analysis (v12.5) demonstrated a high-confidence family score (0.99999) for a low-grade glioneuronal tumor, with the highest methylation class associated with RGNT (0.99998).

The final integrated diagnosis was consistent with RGNT. Now 36 months since diagnosis, initial slow but progressive growth of the tumor has been described, with subsequent stabilization.

RNA-sequencing studies were performed to better understand how these individuals cluster based on similarities in gene expression profiles. A UMAP data structure, as derived from RNA-sequencing studies, was generated from individuals with CNS tumors studied as part of the NCH cohort (*n* = 235) and the UCSC Treehouse Initiative (*n* = 791). Individuals 1 and 2 clustered closely together amid other LGG and glioneuronal tumors. This suggests similarity in gene expression between each tumor which may reflect the shared mutational profile, including activating *FGFR1* variants, in addition to *PIK3R1* variants predicted to activate the PI3K signaling pathway, in concert with the germline *PTPN11* variant (**Supplementary Figure 3**, **Table 2**).

### Individual 3

**Figure 3:**
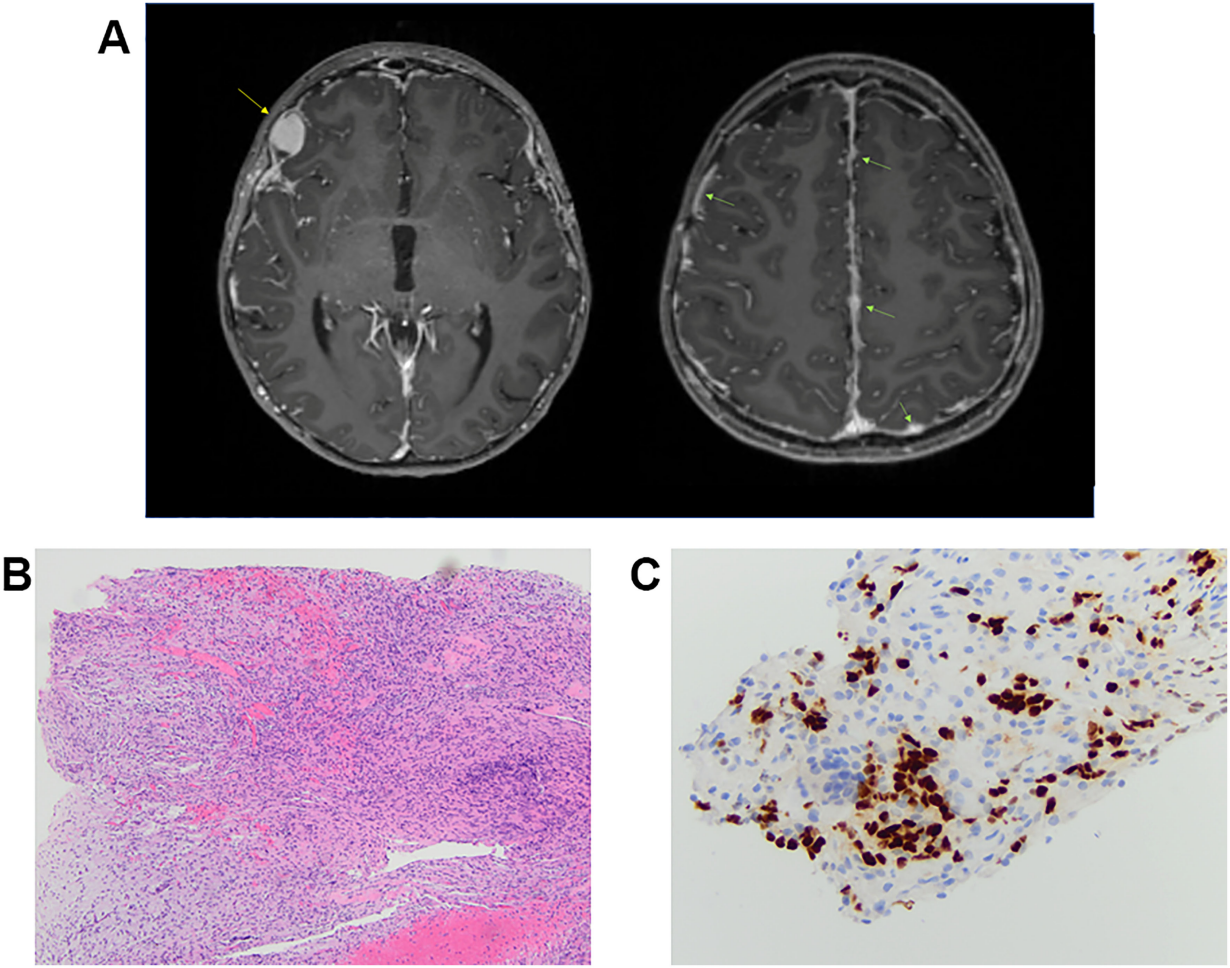
Individual 3—radiologic and histopathologic studies of the tumor. **(A)** Axial T1-weighted, post-contrast images in two planes demonstrate a growing, enhancing right frontal nodule (yellow arrow) as well as nodular leptomeningeal enhancement and thickening throughout (green arrows). **(B)** H&E shows a low-grade-appearing glioneuronal tumor with features suggestive of diffuse leptomeningeal glioneuronal tumor. **(C)** Immunohistochemical stain shows tumor cell immunoreactivity for Olig2 [Olig2, ×40 ocular mag.].

A 9-year-old female patient presented with a transient headache and left-sided weakness which led to a brain and spine MRI being performed. Imaging revealed diffuse dural enhancement and nodular thickening of unknown etiology (without a focal primary mass) for which an oncologic process could not be ruled out. Cerebrospinal fluid cytology analysis following lumbar puncture was negative for malignant cells. Full body positron emission tomography (PET)/computed tomography (CT) scan did not demonstrate obvious extraneural disease. She presented 1 month later and repeat imaging showed persistent linear and nodular leptomeningeal enhancement with progressive increased fluid-attenuated inversion recovery (FLAIR) signal abnormalities in the periventricular and deep white matter of bilateral frontal and parietal lobes (**Figure 3A**). At this time, a dural and leptomeningeal biopsy was performed. Neuropathologic evaluation revealed an unusual glioneuronal tumor with morphologic features suggestive of DLGNT. The tumor was composed of moderately cellular nodules of small bland cells with oval to elongate nuclei embedded in a desmoplastic to collagenous stroma (**Figure 3B**). No definite neuronal component was identified by morphology. Mitoses were not found. The tumor cells were immunoreactive for Olig2 (**Figure 3C**) and GFAP. Synaptophysin immunostain highlighted very rare small cells.

Paired exome analysis of disease-involved tissue (estimated 30% tumor content) and a comparator sample revealed a germline missense variant in *PTPN11* p.Lys70Arg (classified as expert panel pathogenic in ClinVar, variation ID: 44603) (**Table 2**). The tumor harbored a somatic hotspot activating variant in *KRAS* p.Gly12Asp. The somatic copy number analysis demonstrated a focal amplification of 4q12 (encompassing the *PDGFRA*, *KIT*, *KDR*, *CHIC2*, and *FIP1L1* loci), a loss of chromosome arm 1p, and a gain of 1q (**Supplementary Figure 1C**). Targeted fusion analysis was negative, including for *KIAA1549::BRAF*. In support of a DLGNT diagnosis, *IDH1* and *IDH2* were considered wild type with no evidence of mutation. DNA methylation analysis (v12.5) failed to classify confidently to a specific tumor type, with a family score of 0.39560 for meningioma and a class score of 0.35455 for meningioma, benign. The low scores may be attributed to low tumor cellularity in the sample under study. UMAP visualization of gene expression data demonstrated that the tumor from individual 3 clustered uniquely from those of individuals 1 and 2 and instead clustered with a separate group of LGGs (**Supplementary Figure 3**).

Given the unusual molecular findings and methylome features not entirely consistent with typical DLGNT (yet displaying MAPK pathway alteration and 1p loss, with Olig2 positivity and rare cells with synaptophysin immunoreactivity), the tumor was ultimately diagnosed as a glioneuronal neoplasm with worrisome molecular features, NEC. Following the identification of the germline *PTPN11* variant in the translational study, sequencing of this locus was performed at an outside clinical laboratory which confirmed the presence of the variant. A review of the patient’s clinical characteristics and history revealed that she had previously unrecognized features consistent with NS, including short stature, concerns for poor growth, dysmorphic facial features, easy bruising, and atrial septal defect. Notably, identification of this variant led to cascade testing of family members, and this variant was found to be *de novo* in our proband.

The patient was treated with the oral MEK inhibitor trametinib, with an initial decrease in disease burden (at 6 weeks), but then rapid progression of leptomeningeal disease and a new, growing right frontal nodule 2.5 months later. At this time, the decision was made to proceed with proton craniospinal irradiation (CSI; 41.4 Gy CSI with 54 Gy boost to right frontal nodule) given early disease progression on targeted therapy and concern for higher-risk molecular features [1q gain and 4q12 amplification (containing *PDGFRA* and other loci)]. The patient is now 18 months post-completion of proton CSI, with concern for disease progression based on radiographic findings, and has started maintenance chemotherapy with temozolomide and lomustine.

### Individual 4

**Figure 4:**
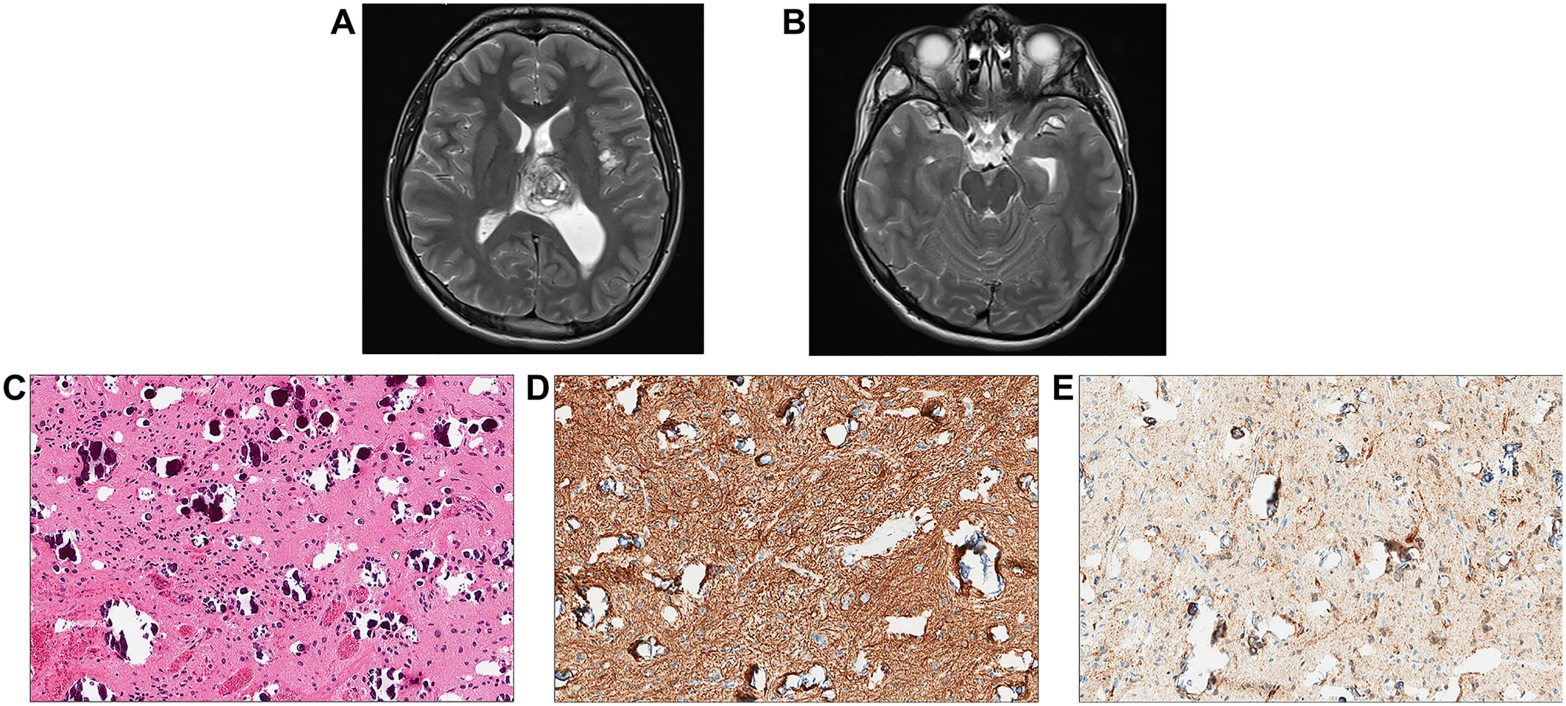
Individual 4- Radiologic and Histopathologic Studies of the Tumor. **(A, B)** Axial T2 contrast-weighted images demonstrate a mass lesion ~3.6 cm in greatest dimension adjacent to the left thalamus within the ventricle. The left insular cortex contains a separate lobular lesion approximately 1 cm in diameter in within the right temporal fossa there is also a soft tissue lesion measuring approximately 3 cm in maximum dimension. **(C)** H&E shows a low-grade appearing glioneuronal tumor with abundant calcifications. **(D)** Immunohistochemical staining for GFAP is positive. **(E)**A subset of cells appears positive for synaptophysin.

An 18-year-old male patient with a history of short stature, cryptorchidism, gastroesophageal reflux, and minor cardiac leaflet abnormalities presented with progressive headaches. Maternal history was positive for benign brain tumors, epilepsy, and mild pulmonary stenosis; however, neither the mother, patient, nor other family members were previously known to have a diagnosis of NS. Brain MRI/magnetic resonance angiography (MRA) found a primary hemorrhagic lesion arising from the left thalamus and filling a portion of the left lateral ventricle, along with two distinct lesions involving the left insular cortex and the right temporal fossa (**Figures 4A, B**). Spinal imaging was negative. Subtotal resection of the primary thalamic lesion and ventriculoperitoneal (VP) shunt placement were performed. Histologic examination of the tumor showed a low-grade glial neoplasm with abundant calcifications (**Figures 4C–E**). No rosettes or pseudorosettes were identified. Foci of microvascular proliferation were noted along with areas of acute hemorrhage.

Targeted molecular profiling of disease-involved tissue and a peripheral blood sample using OPXv7 identified a heterozygous germline pathogenic gain-of-function variant in *PTPN11* p.Asn308Asp (classified as expert panel pathogenic in ClinVar, variation ID: 13326) (**Table 2**), prompting a new diagnosis of NS. The tumor tissue was also positive for well-characterized activating alterations in *FGFR1* p.Asn546Lys and two variants in *PIK3CA* p.His1047Arg and p.His1047Tyr (**Supplementary Figure 4**). No clinically actionable gene fusions or copy number alterations were otherwise identified. The tumor did not demonstrate any evidence of microsatellite instability using the mSINGs method (49), and the tumor mutational burden was low, estimated to be approximately 1 mutation per megabase (50).

The final integrated diagnosis of the tumor was a low-grade glioneuronal tumor, NEC. Although this tumor had histologic and molecular features suggestive of an RGNT, that diagnosis could not be established given the lack of rosettes in an otherwise well-sampled tumor. The additional incidentally found lesion in the insular cortex was determined to be consistent with benign LGG, and biopsy was not indicated. The temporal fossa lesion was determined to be most compatible with a vascular malformation, as has been reported in NS and other RASopathies (2). Postoperatively, this individual developed right-sided hemiparesis and was found to have a venous infarct in the left thalamus and basal ganglia. Routine imaging 3 months following resection found ventriculomegaly with trapping of the temporal horn of the left lateral ventricle, necessitating a secondary VP shunt placement. Now 6 months since diagnosis, he has no evidence of tumor progression.

### Individual 5

**Figure 5:**
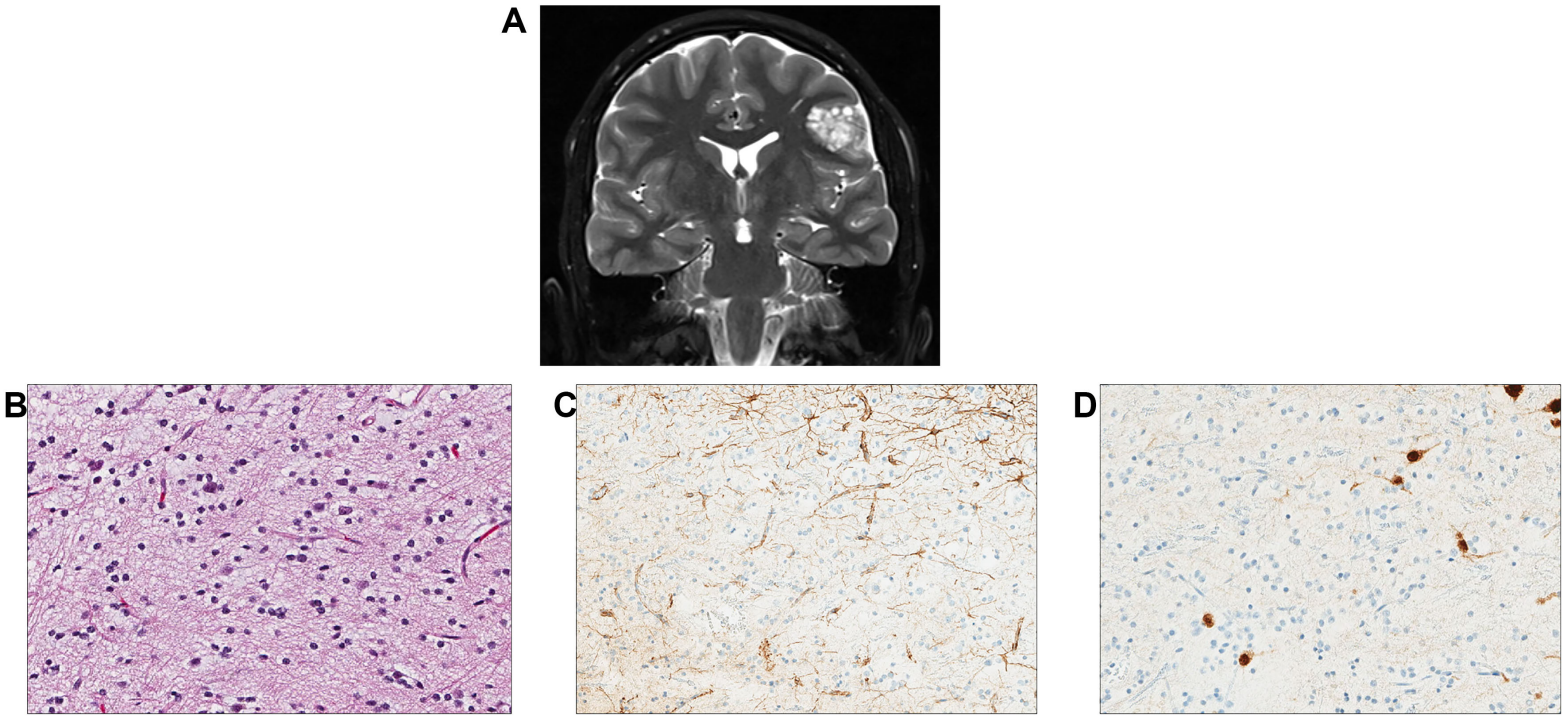
Individual 5—radiologic and histopathologic studies of the tumor. **(A)** A coronal T2 post-contrast image contains a nodular cortically based left frontal mass. **(B)** H&E staining reveals a low-grade glioneuronal tumor with small oligodendroglial-like cells admixed with cytologically normal-appearing neurons. **(C)** A GFAP stain highlights reactive astrocytes but appears negative in the small oligodendroglial-like cells. **(D)** A NeuN stain highlights scattered neurons between the oligodendroglial-like tumor cells.

A 16-year-old male patient had a remote history of growth delay which ultimately led to a workup and diagnosis of NS. He was treated with growth hormone replacement but was otherwise asymptomatic until he developed seizures. A brain MRI identified a left parietal lobe mass measuring 2.2 × 1.8 cm with increased T2/FLAIR signal intensity (**Figure 5A**). The MRI also identified small foci of signal abnormality in the thalamus. Gross total surgical resection of the main tumor mass was performed. Histologically, the tumor was largely composed of small oligodendroglial-like cells with round nuclei with occasional nuclear halos along with scattered interspersed cytologically normal neurons (**Figures 5B–D**). The background had a myxoid appearance in areas though no definitive “floating” neurons were identified. Delicate chicken wire-like vessels were noted in the background.

Molecular analysis of tumor tissue and a peripheral blood sample using OPXv7 identified a heterozygous germline pathogenic gain-of-function variant in *PTPN11* p.Asn58Lys (classified as pathogenic in ClinVar, variation ID: 40488) and a germline heterozygous *EGFR* nonsense variant p.Arg531Ter along with a somatic *FGFR1* p.Asn546Lys alteration (**Table 2**). The *PTPN11* variant conferred a diagnosis of Noonan syndrome. This individual also harbored a germline inactivating variant in *EGFR*, for which he would be a carrier for autosomal recessive neonatal inflammatory skin and bowel disease (51). Clinical methylation profiling of the tumor demonstrated no high confidence match but was suggestive of DNET. The tumor did not demonstrate any evidence of microsatellite instability using the mSINGs method (49), and the tumor mutational burden was low, estimated to be approximately 1 mutation per megabase (50).

The final integrated molecular diagnosis of the tumor was consistent with DNET. Following surgical resection, this individual did well and had no subsequent seizures. Follow-up imaging studies have been stable with no new areas of signal abnormality in radiographic studies.

## Discussion

**Figure 6:**
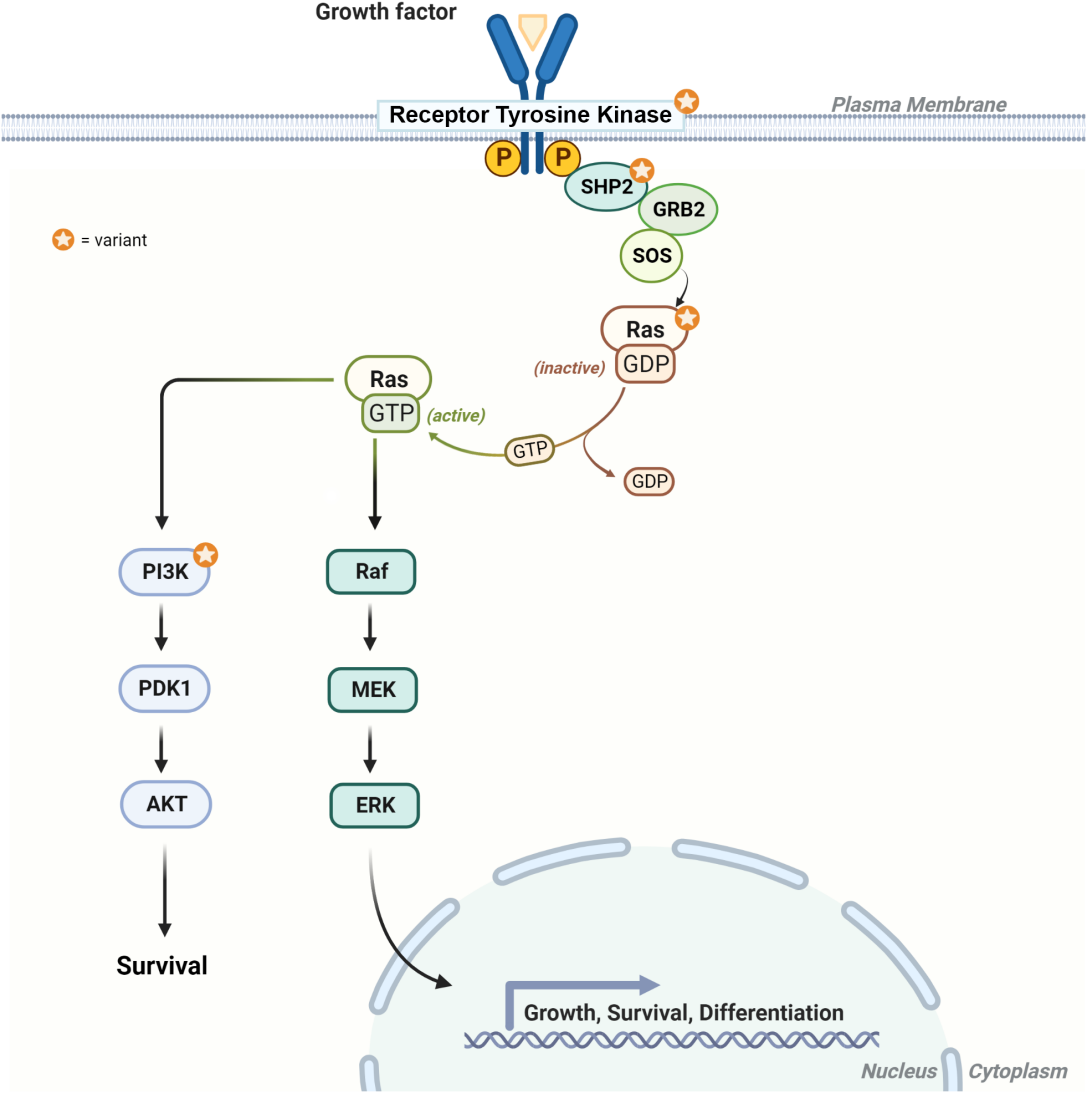
Diagram of the RAS/MAPK and PI3K signaling pathways. The star highlights the protein products with genetic alterations described in individuals 1–5. RTK = receptor tyrosine kinase (e.g., *FGFR1*), SHP2 (protein product for *PTPN11*), Ras (e.g., *KRAS*), and PI3K [heterodimer comprising a catalytic (e.g., *PIK3CA*) and regulatory subunit (*PIK3R1*)].

**Figure 7:**
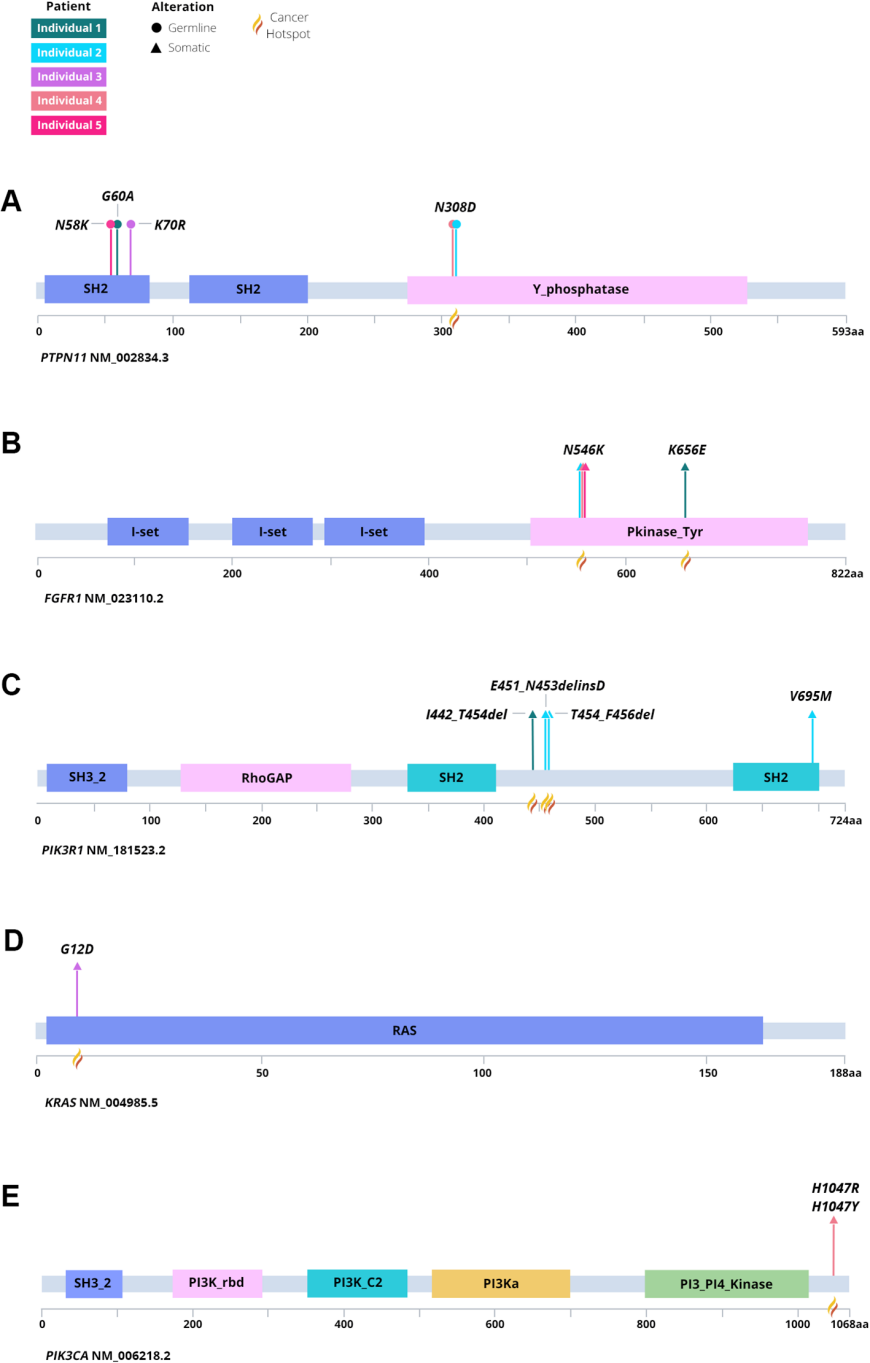
Lollipop plot of germline and somatic variation identified among each individual and tumor. Identified variants in **(A)** *PTPN11*, **(B)** *FGFR1*, **(C)** *PIK3R1*, **(D)** *KRAS*, and **(E)** *PIK3CA* denoting germline or somatic origin.

Herein, we report on the clinical features, histopathologic characteristics, and clinical course associated with molecularly characterized gliomas and glioneuronal tumors among a group of individuals with NS. Part of the RASopathies spectrum, NS is associated with a heightened risk of pediatric malignancies, with a standardized incidence ratio of 8.1 in childhood cancer registries (6, 8). A spectrum of CNS tumors has been reported in association with NS, including LGG, medulloblastoma, HGG, and others (52, 53). *PTPN11* is the most commonly altered gene in NS and encodes the SH2 domain-containing tyrosine phosphatase 2 (SHP-2), modulating the RAS/MAPK and the PI3K/AKT pathways, with resultant control in cell growth and differentiation (**Figure 6**) (54, 55). Dysregulation of these pathways leads to altered cellular proliferation, invasion, and inhibition of apoptosis, contributing to tumorigenesis (56, 57). In this report, we describe the molecular characteristics of gliomas or glioneuronal tumors in five individuals with NS associated with *PTPN11*. All five individuals also had somatic alterations which led to activation of the RAS/MAPK and PI3K/AKT signaling pathways (**Table 2**, **Figures 6**, **7**).

In 2021, the WHO released guidelines for CNS tumors (13), which emphasized the use of an integrated diagnosis, in which the histopathologic features combined with molecular findings support a final diagnosis. Within these guidelines, the use of comprehensive molecular testing, in particular DNA-based methylation classification, can aid in accurately classifying specific tumors or tumor subtypes. Notably, the WHO identifies numerous tumor types, for which methylation profiling has high diagnostic utility (13). DNA-based methylation classification for CNS tumors was described by Capper et al., in which a machine learning-based classifier was developed to calculate the probability of an individual tumor sample’s similarity to one of the 91 methylation classes included in the classifier’s training dataset (47). Among >1,000 tumors evaluated by methylation classification, 76% were in agreement between histopathology and predicted methylation classification. Nevertheless, methylation analysis may not provide diagnostic certainty in all cases due to an atypical methylation pattern in the studied tumor or underrepresented methylation profiles for a given entity within the applied classifier.

The utility of methylation profiling is limited in tumors with diminished disease content. High tumor content (>60%) is often required to achieve high-confidence classifier scores (58, 59). Furthermore, while some tumor types have a characteristic molecular signature (i.e., methylation profile or pattern of somatic variation), there may be histopathologic, clinical, or molecular features that overlap numerous tumor types making a final integrated diagnosis challenging. For example, the results from DNA methylation analysis for the tumor from individual 3 did not confidently classify a specific tumor type. Given this individual’s clinical course, which has required craniospinal irradiation following a poor response to targeted therapy, and now maintenance chemotherapy due to progressive disease, the integrated diagnosis appropriately raised concern for the potentially aggressive nature of the molecular findings in this tumor.

In NS, most brain tumors are described as glial or glioneuronal, with DNETs accounting for 40% of the published cases. Other reported tumors include low-grade glioneuronal tumors, HGGs, and medulloblastoma (5–7, 52, 53, 60–78). Karafin et al. (5) described an early report of RGNT in NS, demonstrating strong pERK immunoreactivity, suggesting MAPK/ERK pathway activation playing a role in tumor development. Given the clinical, histopathologic, and molecular overlap between LGGs, diagnostic characterization can be challenging. As evidence of such, El-Ayadi et al. (52) reported a total of 24 LGGs in the setting of NS, of which only one was diagnosed as RGNT (5). Within this cohort, numerous morphologic mimics of RGNT were documented, including pilocytic astrocytoma (*n* = 4), pilomyxoid astrocytoma (*n* = 1), oligodendroglioma (*n* = 2), low-grade mixed glial–neuronal tumor (*n* = 1), other low-grade astrocytoma (*n* = 3), and DNET (*n* = 9). Histopathologic diagnosis may well be confounded by small biopsies with a high potential for sampling error. A piloid/pilocytic to oligodendroglial-like morphologic component predominates in most classic RGNT, while the characteristic/diagnostic neurocytic rosettes of RGNT may be quite subtle or only focal/sparse. Similarly, molecular characteristics, such as *FGFR1* alterations, may frequently occur in other LGGs (11). Furthermore, the majority of NS-associated LGGs in the cohort described by El-Ayadi et al. presented in the now well-known typical midline location of RGNT and at least one-third were multifocal and/or disseminated, a generally much less frequent, if not rare, phenomenon in other LGG diagnoses. Thus, it appears highly plausible that some of the other LGGs previously described may well represent additional examples of RGNT occurring in NS and, with updated molecular characterization/analysis, might be subject to reclassification.

Molecular profiling in the absence of a paired germline sample can confound variant interpretation, necessitating confirmatory testing for suspected germline findings. If a variant is inferred to be germline, published guidance from the scientific community supports laboratory-recommended follow-up confirmatory testing (79, 80). In the setting of a paired analysis, such as that performed in this case series, the etiology of called variants is clearly delineated and may prompt genetic counseling and cascade testing. Importantly, a paired analysis can identify germline cancer predisposition in those with previously unrecognized clinical features. As a result of genomic profiling following the cancer diagnosis, two of four *PTPN11-*positive individuals within our NCH pediatric/adolescent and young adult (AYA) cancer cohort (individuals 2 and 3) received an NS diagnosis following their cancer diagnosis as a result of paired exome sequencing. Similarly, among the Seattle cohort, individual 4 remained undiagnosed for NS until paired genomic profiling was performed as a result of their CNS malignancy.

While surgical resection remains the standard of care for individuals with pediatric LGG (22, 23, 25, 28), over the past decade, risk-adapted approaches and molecular-directed therapies have refined the management of pediatric LGGs (11, 28), most notably in patients with progressive or relapsing disease. Additionally, Ryall et al. (28) proposed a risk-stratified approach that integrates clinical, imaging, and molecular data to guide appropriate tumor management. In cases of low-risk stratification, conservative approaches would be suitable, while high-risk stratification would necessitate more aggressive interventions (28).

There is increasing experience demonstrating that molecularly targeted therapies, such as RAF (81–83), MEK (84–87), and mTOR inhibitors (88–90), are effective in patients with pediatric LGGs who have failed first-line chemotherapy; these studies have early results with reassuring short-term safety data. The results of both phase I and II clinical trials have led the efforts to evaluate selumetinib as frontline therapy for pediatric LGGs with (NCT03871257) or without neurofibromatosis-1 (NCT04166409). Additionally, the combination of the BRAF inhibitor dabrafenib and the MEK inhibitor trametinib has recently gained FDA approval as a first-line treatment for pediatric patients with LGG (29), and tovorafenib was recently FDA-approved for use in *BRAF-*altered relapsed or refractory pediatric LGG (91, 92).

Somatic alterations in *FGFR1* and PI3K pathway genes in LGG resulting in upregulation of the RAS/MAPK and the PI3K/AKT/mTOR pathways provide attractive therapeutic targets in the setting of progressive disease. A recent case report describes the use of the mTOR inhibitor everolimus in RGNT which demonstrated radiographic response, after failing first-line treatment with chemotherapy (93). A single-center study observed objective clinical and radiographic responses to the oral FGFR1 inhibitor Debio1347 in three pediatric patients with progressive LGGs, with an acceptable toxicity profile (94). Interestingly, the authors identified specific *FGFR1* alterations that could be associated with improved response, as well as concomitant co-occurrence of *NF1* deletion as a potential mechanism for acquired resistance to therapy. Currently, the FGFR1 inhibitor erdafitinib is under evaluation in children with advanced solid tumors (NCT03210714). In the setting of NS and glioma, the utility of targeted therapy may be challenging, given the activation of the RAS/MAPK and PI3K/AKT pathways at multiple levels throughout the pathway (**Figure 6**). Combinatorial approaches or targeting downstream of SHP-2 (protein product of *PTPN11*) and the PI3K complex, by using MEK and/or mTOR inhibitors, respectively, may be necessary. Targeting RAS genes has proven more difficult due to the lack of binding pockets on the surface of these proteins for targeted therapies to bind; however, studies are ongoing to overcome these challenges (95, 96). Recently, in the setting of lung cancer, advances in RAS variant-specific targeting have been achieved with FDA approval of sotorasib and adagrasib in patients with locally advanced or metastatic non-small cell lung cancer harboring a *KRAS* p.Gly12Cys variant (97, 98).

Our study has several limitations. First, the cohort size and composition limits the generalizability of our findings. For the NCH cohort, individuals with rare or treatment-refractory cancers were nominated for translational research testing by a clinician engaged in their care (99). Thus, selection bias may have influenced those nominated for enrollment on the basis of clinical interest and timing of clinical care. Furthermore, those with low-grade CNS tumors that respond well to surgery or conventional chemotherapy may not have been considered for paired exome sequencing at the time of the study. While we were able to extend the NCH cohort findings through the inclusion of two individuals clinically tested as part of the Seattle cohort, there were fundamental differences between each cohort, and it was not possible to glean an overall frequency of NS in the setting of glioma or glioneuronal tumor amid the available data. Future studies with larger cohorts of individuals with CNS malignancies undergoing comprehensive molecular testing are needed to validate and extend our findings.

In the setting of cancer, comprehensive molecular profiling has demonstrated utility in aiding diagnosis, identifying treatment options, and prognostication. Importantly, utilization of paired tumor and germline samples allows for the identification of germline cancer predisposition variants, the frequency of which has been documented in the range of 10–18% amid pediatric/AYA cancer cohorts (100–102). In a CNS cancer-focused cohort comprised of pediatric/AYA individuals, germline genetic variation associated with cancer susceptibility was identified in 16% (99). Continued broad molecular characterization will extend our understanding of germline cancer susceptibility, particularly the breadth of cancer diagnoses, disease risk, correlation with age, and other genotype–phenotype relationships. Such knowledge potentiates improvement in surveillance recommendations and extension of treatment paradigms. Our study shows that amid this cohort, the characterization of disease was strengthened through the rendering of an integrated diagnosis, supported by multiple molecular testing modalities. This study extends the benefit of comprehensive profiling to aid in both germline and cancer diagnoses, as well as treatment considerations of pediatric/AYA gliomas and glioneuronal tumors.

## Data Availability

The genomic datasets generated and/or analyzed during the current study are available for individuals 1 and 2 in the dbGaP under phs001820.v3.p1 (IGMCH0035 [individual 1]) and IGMCH0125 [individual 2]).

## References

[1] Roberts AE Allanson JE Tartaglia M Gelb BD. Noonan syndrome. Lancet. (2013) 381:333–42. doi: 10.1016/S0140-6736(12)61023-X PMC4267483 23312968

[2] Rauen KA. The RASopathies. Annu Rev Genomics Hum Genet. (2013) 14:355–69. doi: 10.1146/annurev-genom-091212-153523 PMC4115674 23875798

[3] Grant AR Cushman BJ Cave H Dillon MW Gelb BD Gripp KW. Assessing the gene-disease association of 19 genes with the RASopathies using the ClinGen gene curation framework. Hum Mutat. (2018) 39:1485–93. doi: 10.1002/humu.23624 PMC6326381 30311384

[4] Tartaglia M Kalidas K Shaw A Song X Musat DL van der Burgt I. PTPN11 mutations in Noonan syndrome: molecular spectrum, genotype-phenotype correlation, and phenotypic heterogeneity. Am J Hum Genet. (2002) 70:1555–63. doi: 10.1086/340847 PMC379142 11992261

[5] Karafin M Jallo GI Ayars M Eberhart CG Rodriguez FJ. Rosette forming glioneuronal tumor in association with Noonan syndrome: pathobiological implications. Clin Neuropathol. (2011) 30:297–300. doi: 10.5414/NP300374 22011734 PMC3657471

[6] Kratz CP Franke L Peters H Kohlschmidt N Kazmierczak B Finckh U. Cancer spectrum and frequency among children with Noonan, Costello, and cardio-facio-cutaneous syndromes. Br J Cancer. (2015) 112:1392–7. doi: 10.1038/bjc.2015.75 PMC4402457 25742478

[7] Sherman CB Ali-Nazir A Gonzales-Gomez I Finlay JL Dhall G. Primary mixed glioneuronal tumor of the central nervous system in a patient with noonan syndrome: a case report and review of the literature. J Pediatr Hematol Oncol. (2009) 31:61–4. doi: 10.1097/MPH.0b013e31818ab2cf 19125092

[8] Smpokou P Zand DJ Rosenbaum KN Summar ML. Malignancy in Noonan syndrome and related disorders. Clin Genet. (2015) 88:516–22. doi: 10.1111/cge.12568 25683281

[9] Villani A Greer MC Kalish JM Nakagawara A Nathanson KL Pajtler KW. Recommendations for cancer surveillance in individuals with RASopathies and other rare genetic conditions with increased cancer risk. Clin Cancer Res. (2017) 23:e83–90. doi: 10.1158/1078-0432.CCR-17-0631 28620009

[10] Lin FY Bergstrom K Person R Bavle A Ballester LY Scollon S. Integrated tumor and germline whole-exome sequencing identifies mutations in MAPK and PI3K pathway genes in an adolescent with rosette-forming glioneuronal tumor of the fourth ventricle. Cold Spring Harb Mol Case Stud. (2016) 2:a001057. doi: 10.1101/mcs.a001057 27626068 PMC5002928

[11] Ryall S Zapotocky M Fukuoka K Nobre L Guerreiro Stucklin A Bennett J. Integrated molecular and clinical analysis of 1,000 pediatric low-grade gliomas. Cancer Cell. (2020) 37:569–83.e5. doi: 10.1016/j.ccell.2020.03.011 32289278 PMC7169997

[12] Sievers P Appay R Schrimpf D Stichel D Reuss DE Wefers AK. Rosette-forming glioneuronal tumors share a distinct DNA methylation profile and mutations in FGFR1, with recurrent co-mutation of PIK3CA and NF1. Acta Neuropathol. (2019) 138:497–504. doi: 10.1007/s00401-019-02038-4 31250151

[13] Louis DN Perry A Wesseling P Brat DJ Cree IA Figarella-Branger D. The 2021 WHO classification of tumors of the central nervous system: a summary. Neuro Oncol. (2021) 23:1231–51. doi: 10.1093/neuonc/noab106 PMC8328013 34185076

[14] Anyanwu CT Robinson TM Huang JH. Rosette-forming glioneuronal tumor: an update. Clin Transl Oncol. (2020) 22:623–30. doi: 10.1007/s12094-019-02179-8 31313067

[15] Hsu C Kwan G Lau Q Bhuta S. Rosette-forming glioneuronal tumour: imaging features, histopathological correlation and a comprehensive review of literature. Br J Neurosurg. (2012) 26:668–73. doi: 10.3109/02688697.2012.655808 22512825

[16] Pimentel J Resende M Vaz A Reis AM Campos A Carvalho H. Rosette-forming glioneuronal tumor: pathology case report. Neurosurgery. (2008) 62:E1162–3. doi: 10.1227/01.neu.0000325879.75376.63 18580784

[17] Hamauchi S Tanino M Hida K Sasamori T Yano S Tanaka S. Spinal rosette-forming glioneuronal tumor: A case report. Med (Baltimore). (2019) 98:e18271. doi: 10.1097/MD.0000000000018271 PMC6919525 31804365

[18] Yang C Fang J Li G Li S Ha T Wang J. Histopathological, molecular, clinical and radiological characterization of rosette-forming glioneuronal tumor in the central nervous system. Oncotarget. (2017) 8:109175–90. doi: 10.18632/oncotarget.v8i65 PMC5752512 29312599

[19] Appay R Bielle F Sievers P Barets D Fina F Boutonnat J. Rosette-forming glioneuronal tumours are midline, FGFR1-mutated tumours. Neuropathol Appl Neurobiol. (2022) 48:e12813. doi: 10.1111/nan.12813 35293634

[20] Lucas CG Gupta R Doo P Lee JC Cadwell CR Ramani B. Comprehensive analysis of diverse low-grade neuroepithelial tumors with FGFR1 alterations reveals a distinct molecular signature of rosette-forming glioneuronal tumor. Acta Neuropathol Commun. (2020) 8:151. doi: 10.1186/s40478-020-01027-z 32859279 PMC7456392

[21] Louis DN Perry A Reifenberger G von Deimling A Figarella-Branger D Cavenee WK. The 2016 world health organization classification of tumors of the central nervous system: a summary. Acta Neuropathol. (2016) 131:803–20. doi: 10.1007/s00401-016-1545-1 27157931

[22] Beuriat PA Tauziede-Espariat A Pages M Varlet P Di Rocco F. Rosette-forming glioneuronal tumor outside the fourth ventricle: a case-based update. Childs Nerv Syst. (2016) 32:65–8. doi: 10.1007/s00381-015-2922-0 26438552

[23] Hakan T Aker FV. Rosette-forming glioneuronal tumour of the fourth ventricle: case report and review of the literature. Folia Neuropathol. (2016) 54:80–7. doi: 10.5114/fn.2016.58919 27179225

[24] Palejwala AH O'Neal CM Quinton MR Battiste JD Peterson JEG Dunn IF. Polymorphous low-grade neuroepithelial tumor of the young: Rare tumor and review of the literature. Rare Tumors. (2022) 14:20363613221083360. doi: 10.1177/20363613221083360 35371417 PMC8966082

[25] Ramos AA Vega IF Batista KP Fernandez VM Sanchez CR Vega MAA. Rosette-forming glioneuronal tumour of the fourth ventricle. Not always a foreseeable development. Contemp Oncol (Pozn). (2018) 22:270–4. doi: 10.5114/wo.2018.81750 PMC6377414 30783393

[26] Manoharan N Liu KX Mueller S Haas-Kogan DA Bandopadhayay P. Pediatric low-grade glioma: Targeted therapeutics and clinical trials in the molecular era. Neoplasia. (2023) 36:100857. doi: 10.1016/j.neo.2022.100857 36566593 PMC9803951

[27] Mistry M Zhukova N Merico D Rakopoulos P Krishnatry R Shago M. BRAF mutation and CDKN2A deletion define a clinically distinct subgroup of childhood secondary high-grade glioma. J Clin Oncol. (2015) 33:1015–22. doi: 10.1200/JCO.2014.58.3922 PMC4356711 25667294

[28] Ryall S Tabori U Hawkins C. Pediatric low-grade glioma in the era of molecular diagnostics. Acta Neuropathol Commun. (2020) 8:30. doi: 10.1186/s40478-020-00902-z 32164789 PMC7066826

[29] Barbato MI Nashed J Bradford D Ren Y Khasar S Miller CP. FDA approval summary: dabrafenib in combination with trametinib for BRAFV600E mutation-positive low-grade glioma. Clin Cancer Res. (2024) 30:263–8. doi: 10.1158/1078-0432.CCR-23-1503 PMC10841289 37610803

[30] Mackay A Burford A Carvalho D Izquierdo E Fazal-Salom J Taylor KR. Integrated molecular meta-analysis of 1,000 pediatric high-grade and diffuse intrinsic pontine glioma. Cancer Cell. (2017) 32:520–37.e5. doi: 10.1016/j.ccell.2017.08.017 28966033 PMC5637314

[31] Kulubya ES Kercher MJ Phillips HW Antony R Edwards MSB. Advances in the treatment of pediatric brain tumors. Children (Basel). (2022) 10 (1):62. doi: 10.3390/children10010062 36670613 PMC9856380

[32] Kelly BJ Fitch JR Hu Y Corsmeier DJ Zhong H Wetzel AN. Churchill: an ultra-fast, deterministic, highly scalable and balanced parallelization strategy for the discovery of human genetic variation in clinical and population-scale genomics. Genome Biol. (2015) 16:6. doi: 10.1186/s13059-014-0577-x 25600152 PMC4333267

[33] Van der Auwera G O'Connor B. Genomics in the Cloud: Using Docker, GATK, and WDL in Terra. 1st Edition. Sebastopol, CA, United States: O'Reilly Media, Inc (2020).

[34] Poplin R Ruano-Rubio V DePristo M Fennell T Carneiro M van der Auwera G. Scaling accurate genetic variant discovery to tens of thousands of samples. BioRxiv. (2018). doi: 10.1101/201178

[35] Cibulskis K Lawrence MS Carter SL Sivachenko A Jaffe D Sougnez C. Sensitive detection of somatic point mutations in impure and heterogeneous cancer samples. Nat Biotechnol. (2013) 31:213–9. doi: 10.1038/nbt.2514 PMC3833702 23396013

[36] Zhang J Walsh MF Wu G Edmonson MN Gruber TA Easton J. Germline mutations in predisposition genes in pediatric cancer. N Engl J Med. (2015) 373:2336–46. doi: 10.1056/NEJMoa1508054 PMC4734119 26580448

[37] Sondka Z Bamford S Cole CG Ward SA Dunham I Forbes SA. The COSMIC Cancer Gene Census: describing genetic dysfunction across all human cancers. Nat Rev Cancer. (2018) 18:696–705. doi: 10.1038/s41568-018-0060-1 30293088 PMC6450507

[38] Koboldt DC Zhang Q Larson DE Shen D McLellan MD Lin L. VarScan 2: somatic mutation and copy number alteration discovery in cancer by exome sequencing. Genome Res. (2012) 22:568–76. doi: 10.1101/gr.129684.111 PMC3290792 22300766

[39] LaHaye S Fitch JR Voytovich KJ Herman AC Kelly BJ Lammi GE. Discovery of clinically relevant fusions in pediatric cancer. BMC Genomics. (2021) 22:872. doi: 10.1186/s12864-021-08094-z 34863095 PMC8642973

[40] Haas BJ Dobin A Li B Stransky N Pochet N Regev A. Accuracy assessment of fusion transcript detection via read-mapping and *de novo* fusion transcript assembly-based methods. Genome Biol. (2019) 20:213. doi: 10.1186/s13059-019-1842-9 31639029 PMC6802306

[41] Wang K Singh D Zeng Z Coleman SJ Huang Y Savich GL. MapSplice: accurate mapping of RNA-seq reads for splice junction discovery. Nucleic Acids Res. (2010) 38:e178. doi: 10.1093/nar/gkq622 20802226 PMC2952873

[42] Nicorici D Satalan M Edgren H Kangaspeska S Murumagi A Kallioniemi O. FusionCatcher - a tool for finding somatic fusion genes in paired-end RNA-sequencing data. bioRxiv. (2014). doi: 10.1101/011650

[43] Ge H Liu K Juan T Fang F Newman M Hoeck W. FusionMap: detecting fusion genes from next-generation sequencing data at base-pair resolution. Bioinformatics. (2011) 27:1922–8. doi: 10.1093/bioinformatics/btr310 21593131

[44] Davidson NM Majewski IJ Oshlack A. JAFFA: High sensitivity transcriptome-focused fusion gene detection. Genome Med. (2015) 7:43. doi: 10.1186/s13073-015-0167-x 26019724 PMC4445815

[45] Tian L Li Y Edmonson MN Zhou X Newman S McLeod C. CICERO: a versatile method for detecting complex and diverse driver fusions using cancer RNA sequencing data. Genome Biol. (2020) 21:126. doi: 10.1186/s13059-020-02043-x 32466770 PMC7325161

[46] Uhrig S Ellermann J Walther T Burkhardt P Frohlich M Hutter B. Accurate and efficient detection of gene fusions from RNA sequencing data. Genome Res. (2021) 31:448–60. doi: 10.1101/gr.257246.119 PMC7919457 33441414

[47] Capper D Jones DTW Sill M Hovestadt V Schrimpf D Sturm D. DNA methylation-based classification of central nervous system tumours. Nature. (2018) 555:469–74. doi: 10.1038/nature26000 PMC6093218 29539639

[48] Kuo AJ Paulson VA Hempelmann JA Beightol M Todhunter S Colbert BG. Validation and implementation of a modular targeted capture assay for the detection of clinically significant molecular oncology alterations. Pract Lab Med. (2020) 19:e00153. doi: 10.1016/j.plabm.2020.e00153 32123717 PMC7038441

[49] Salipante SJ Scroggins SM Hampel HL Turner EH Pritchard CC. Microsatellite instability detection by next generation sequencing. Clin Chem. (2014) 60:1192–9. doi: 10.1373/clinchem.2014.223677 24987110

[50] Chalmers ZR Connelly CF Fabrizio D Gay L Ali SM Ennis R. Analysis of 100,000 human cancer genomes reveals the landscape of tumor mutational burden. Genome Med. (2017) 9:34. doi: 10.1186/s13073-017-0424-2 28420421 PMC5395719

[51] Campbell P Morton PE Takeichi T Salam A Roberts N Proudfoot LE. Epithelial inflammation resulting from an inherited loss-of-function mutation in EGFR. J Invest Dermatol. (2014) 134:2570–8. doi: 10.1038/jid.2014.164 PMC4090136 24691054

[52] El-Ayadi M Ansari M Kuhnol CD Bendel A Sturm D Pietsch T. Occurrence of high-grade glioma in Noonan syndrome: Report of two cases. Pediatr Blood Cancer. (2019) 66:e27625. doi: 10.1002/pbc.27625 30693642

[53] Lodi M Boccuto L Carai A Cacchione A Miele E Colafati GS. Low-grade gliomas in patients with noonan syndrome: case-based review of the literature. Diagnostics (Basel). (2020) 10 (8):582. doi: 10.3390/diagnostics10080582 32806529 PMC7460327

[54] Tajan M Paccoud R Branka S Edouard T Yart A. The RASopathy family: consequences of germline activation of the RAS/MAPK pathway. Endocr Rev. (2018) 39:676–700. doi: 10.1210/er.2017-00232 29924299

[55] Tidyman WE Rauen KA. The RASopathies: developmental syndromes of Ras/MAPK pathway dysregulation. Curr Opin Genet Dev. (2009) 19:230–6. doi: 10.1016/j.gde.2009.04.001 PMC2743116 19467855

[56] Burotto M Chiou VL Lee JM Kohn EC. The MAPK pathway across different Malignancies: a new perspective. Cancer. (2014) 120:3446–56. doi: 10.1002/cncr.28864 PMC4221543 24948110

[57] De Luca A Maiello MR D'Alessio A Pergameno M Normanno N. The RAS/RAF/MEK/ERK and the PI3K/AKT signalling pathways: role in cancer pathogenesis and implications for therapeutic approaches. Expert Opin Ther Targets. (2012) 16 Suppl 2:S17–27. doi: 10.1517/14728222.2011.639361 22443084

[58] Capper D Stichel D Sahm F Jones DTW Schrimpf D Sill M. Practical implementation of DNA methylation and copy-number-based CNS tumor diagnostics: the Heidelberg experience. Acta Neuropathol. (2018) 136:181–210. doi: 10.1007/s00401-018-1879-y 29967940 PMC6060790

[59] Priesterbach-Ackley LP Boldt HB Petersen JK Bervoets N Scheie D Ulhoi BP. Brain tumour diagnostics using a DNA methylation-based classifier as a diagnostic support tool. Neuropathol Appl Neurobiol. (2020) 46:478–92. doi: 10.1111/nan.12610 PMC7496466 32072658

[60] Selter M Dresel R Althaus J Baz Bartels M Dittrich S Geb S. Dysembryoplastic neuroepithelial tumor (DNET) in a patient with Noonan syndrome. Abstracts of the 36th Annual Meeting of the Society of Neuropediatrics; Mannheim, Germany. Neuropediatrics. (2010) 41:1356. doi: 10.1055/s-0030-1265602

[61] Bangalore Krishna K Pagan P Escobar O Popovic J. Occurrence of cranial neoplasms in pediatric patients with noonan syndrome receiving growth hormone: is screening with brain MRI prior to initiation of growth hormone indicated? Horm Res Paediatr. (2017) 88:423–6. doi: 10.1159/000479107 PMC9204260 28746941

[62] Bendel A Hansen M Dugan S Mendelsohn N. Dysembryoplastic neuroepithelial tumor in two relatives with Noonan syndrome and a PTPN11 mutation. Neuro-Oncology. (2012). 14 (suppl_1): i148–56. doi: 10.1093/neuonc/nos108

[63] Charlton ME Stitzenberg KB Lin C Schlichting JA Halfdanarson TR Juarez GY. Predictors of long-term quality of life for survivors of stage II/III rectal cancer in the cancer care outcomes research and surveillance consortium. J Oncol Pract. (2015) 11:e476–86. doi: 10.1200/JOP.2015.004564 PMC4507395 26080831

[64] de Jong M Schieving J Goraj B. Remarkable intra-cerebral lesions on MRI in a patient with Noonan syndrome. Eur J Radiol Extra. (2011) 78:e17–e9. doi: 10.1016/j.ejrex.2011.01.005

[65] Delisle M Siegfried A Tauber M Cave H Loukh N Boetto S. Dysembryoplastic neuroepithelial tumor (DNET) and Noonan syndrome. A case report. Brain Pathol. (2014). doi: 10.1111/bpa.12184

[66] Fryssira H Leventopoulos G Psoni S Kitsiou-Tzeli S Stavrianeas N Kanavakis E. Tumor development in three patients with Noonan syndrome. Eur J Pediatr. (2008) 167:1025–31. doi: 10.1007/s00431-007-0636-3 18057963

[67] Jongmans M Sistermans EA Rikken A Nillesen WM Tamminga R Patton M. Genotypic and phenotypic characterization of Noonan syndrome: new data and review of the literature. Am J Med Genet A. (2005) 134A:165–70. doi: 10.1002/(ISSN)1552-4833 15723289

[68] Jongmans MC van der Burgt I Hoogerbrugge PM Noordam K Yntema HG Nillesen WM. Cancer risk in patients with Noonan syndrome carrying a PTPN11 mutation. Eur J Hum Genet. (2011) 19:870–4. doi: 10.1038/ejhg.2011.37 PMC3172922 21407260

[69] Martinelli S Carta C Flex E Binni F Cordisco EL Moretti S. Activating PTPN11 mutations play a minor role in pediatric and adult solid tumors. Cancer Genet Cytogenet. (2006) 166:124–9. doi: 10.1016/j.cancergencyto.2005.10.003 16631468

[70] McWilliams GD SantaCruz K Hart B Clericuzio C. Occurrence of DNET and other brain tumors in Noonan syndrome warrants caution with growth hormone therapy. Am J Med Genet A. (2016) 170A:195–201. doi: 10.1002/ajmg.a.37379 26377682

[71] Nair S Fort JA Yachnis AT Williams CA. Optic nerve pilomyxoid astrocytoma in a patient with Noonan syndrome. Pediatr Blood Cancer. (2015) 62:1084–6. doi: 10.1002/pbc.25382 25585602

[72] Pellegrin MC Tornese G Cattaruzzi E Blank E Kieslich M Ventura A eds. A rare brain tumor in noonan syndrome: Report of two cases. Dublin, Ireland: European Society for Paediatric Endocrinology (2014).

[73] Rankin J Short J Turnpenny P Castle B Hanemann CO. Medulloblastoma in a patient with the PTPN11 p.Thr468Met mutation. Am J Med Genet A. (2013) 161A:2027–9. doi: 10.1002/ajmg.a.36005 23813970

[74] Rush S Madden J Hemenway M Foreman N. Mutations in PTPN11 gene may predispose to development of midline low grade gliomas. Neuro Oncol. (2014) 16(Suppl 1):i60–i70.

[75] Sanford RA Bowman R Tomita T De Leon G Palka P. A 16-year-old male with Noonan's syndrome develops progressive scoliosis and deteriorating gait. Pediatr Neurosurg. (1999) 30:47–52. doi: 10.1159/000028761 10202309

[76] Schuettpelz LG McDonald S Whitesell K Desruisseau DM Grange DK Gurnett CA. Pilocytic astrocytoma in a child with Noonan syndrome. Pediatr Blood Cancer. (2009) 53:1147–9. doi: 10.1002/pbc.22193 19621452

[77] Siegfried A Cances C Denuelle M Loukh N Tauber M Cave H. Noonan syndrome, PTPN11 mutations, and brain tumors. A clinical report and review of the literature. Am J Med Genet A. (2017) 173:1061–5. doi: 10.1002/ajmg.a.38108 28328117

[78] Takagi M Miyashita Y Koga M Ebara S Arita N Kasayama S. Estrogen deficiency is a potential cause for osteopenia in adult male patients with Noonan's syndrome. Calcif Tissue Int. (2000) 66:200–3. doi: 10.1007/s002230010040 10666495

[79] Raymond VM Gray SW Roychowdhury S Joffe S Chinnaiyan AM Parsons DW. Germline findings in tumor-only sequencing: points to consider for clinicians and laboratories. J Natl Cancer Inst. (2015) 108 (4):djv351. doi: 10.1093/jnci/djv351 26590952 PMC4849259

[80] Li MM Chao E Esplin ED Miller DT Nathanson KL Plon SE. Points to consider for reporting of germline variation in patients undergoing tumor testing: a statement of the American College of Medical Genetics and Genomics (ACMG). Genet Med. (2020) 22:1142–8. doi: 10.1038/s41436-020-0783-8 32321997

[81] Hargrave DR Bouffet E Tabori U Broniscer A Cohen KJ Hansford JR. Efficacy and safety of dabrafenib in pediatric patients with BRAF V600 mutation-positive relapsed or refractory low-grade glioma: results from a phase I/IIa study. Clin Cancer Res. (2019) 25:7303–11. doi: 10.1158/1078-0432.CCR-19-2177 31811016

[82] Kieran MW Geoerger B Dunkel IJ Broniscer A Hargrave D Hingorani P. A phase I and pharmacokinetic study of oral dabrafenib in children and adolescent patients with recurrent or refractory BRAF V600 mutation-positive solid tumors. Clin Cancer Res. (2019) 25:7294–302. doi: 10.1158/1078-0432.CCR-17-3572 31506385

[83] Nicolaides T Nazemi KJ Crawford J Kilburn L Minturn J Gajjar A. Phase I study of vemurafenib in children with recurrent or progressive BRAF(V600E) mutant brain tumors: Pacific Pediatric Neuro-Oncology Consortium study (PNOC-002). Oncotarget. (2020) 11:1942–52. doi: 10.18632/oncotarget.v11i21 PMC7260122 32523649

[84] Banerjee A Jakacki RI Onar-Thomas A Wu S Nicolaides T Young Poussaint T. A phase I trial of the MEK inhibitor selumetinib (AZD6244) in pediatric patients with recurrent or refractory low-grade glioma: a Pediatric Brain Tumor Consortium (PBTC) study. Neuro Oncol. (2017) 19:1135–44. doi: 10.1093/neuonc/now282 PMC5570236 28339824

[85] Fangusaro J Onar-Thomas A Poussaint TY Wu S Ligon AH Lindeman N. A phase II trial of selumetinib in children with recurrent optic pathway and hypothalamic low-grade glioma without NF1: a Pediatric Brain Tumor Consortium study. Neuro Oncol. (2021) 23:1777–88. doi: 10.1093/neuonc/noab047 PMC8485450 33631016

[86] Fangusaro J Onar-Thomas A Young Poussaint T Wu S Ligon AH Lindeman N. Selumetinib in paediatric patients with BRAF-aberrant or neurofibromatosis type 1-associated recurrent, refractory, or progressive low-grade glioma: a multicentre, phase 2 trial. Lancet Oncol. (2019) 20:1011–22. doi: 10.1016/S1470-2045(19)30277-3 PMC6628202 31151904

[87] Selt F van Tilburg CM Bison B Sievers P Harting I Ecker J. Response to trametinib treatment in progressive pediatric low-grade glioma patients. J Neurooncol. (2020) 149:499–510. doi: 10.1007/s11060-020-03640-3 33026636 PMC7609413

[88] Mueller S Aboian M Nazemi K Gauvain K Yoon J Minturn J. LGG-53. PNOC001 (NCT01734512): a phase ii study of everolimus for recurrent or progressive pediatric low-grade gliomas (pLGG). Neuro-Oncology. (2020) 22:iii376. doi: 10.1093/neuonc/noaa222.431

[89] Ullrich NJ Prabhu SP Reddy AT Fisher MJ Packer R Goldman S. A phase II study of continuous oral mTOR inhibitor everolimus for recurrent, radiographic-progressive neurofibromatosis type 1-associated pediatric low-grade glioma: a Neurofibromatosis Clinical Trials Consortium study. Neuro Oncol. (2020) 22:1527–35. doi: 10.1093/neuonc/noaa071 PMC7566451 32236425

[90] Wright KD Yao X London WB Kao PC Gore L Hunger S. A POETIC Phase II study of continuous oral everolimus in recurrent, radiographically progressive pediatric low-grade glioma. Pediatr Blood Cancer. (2021) 68:e28787. doi: 10.1002/pbc.28787 33140540 PMC9161236

[91] van Tilburg CM Kilburn LB Perreault S Schmidt R Azizi AA Cruz-Martinez O. LOGGIC/FIREFLY-2: a phase 3, randomized trial of tovorafenib vs. chemotherapy in pediatric and young adult patients with newly diagnosed low-grade glioma harboring an activating RAF alteration. BMC Cancer. (2024) 24:147. doi: 10.1186/s12885-024-11820-x 38291372 PMC10826080

[92] Kilburn LB Khuong-Quang DA Hansford JR Landi D van der Lugt J Leary SES. The type II RAF inhibitor tovorafenib in relapsed/refractory pediatric low-grade glioma: the phase 2 FIREFLY-1 trial. Nat Med. (2024) 30:207–17. doi: 10.1038/s41591-023-02668-y PMC10803270 37978284

[93] McNall-Knapp R Gross N Zaky W Cornwell B Gavula T Bavle A. LGG-47. systemic therapy of rosette-forming glioneuronal tumor of the fourth ventricle inan adolescent. Neuro-Oncology. (2020) 22:iii375. doi: 10.1093/neuonc/noaa222.425

[94] Farouk Sait S Gilheeney SW Bale TA Haque S Dinkin MJ Vitolano S. Debio1347, an oral FGFR inhibitor: results from a single-center study in pediatric patients with recurrent or refractory FGFR-altered gliomas. JCO Precis Oncol. (2021) 5:PO.20.00444. doi: 10.1200/PO.20.00444 PMC8232545 34250399

[95] Chen K Zhang Y Qian L Wang P. Emerging strategies to target RAS signaling in human cancer therapy. J Hematol Oncol. (2021) 14:116. doi: 10.1186/s13045-021-01127-w 34301278 PMC8299671

[96] Moore AR Rosenberg SC McCormick F Malek S. RAS-targeted therapies: is the undruggable drugged? Nat Rev Drug Discovery. (2020) 19:533–52. doi: 10.1038/s41573-020-0068-6 PMC7809886 32528145

[97] Janne PA Riely GJ Gadgeel SM Heist RS Ou SI Pacheco JM. Adagrasib in non-small-cell lung cancer harboring a KRAS(G12C) mutation. N Engl J Med. (2022) 387:120–31. doi: 10.1056/NEJMoa2204619 35658005

[98] Skoulidis F Li BT Dy GK Price TJ Falchook GS Wolf J. Sotorasib for lung cancers with KRAS p.G12C mutation. N Engl J Med. (2021) 384:2371–81. doi: 10.1056/NEJMoa2103695 PMC9116274 34096690

[99] Mardis ER Potter SL Schieffer KM Varga EA Mathew MT Costello HM. Germline susceptibility from broad genomic profiling of pediatric brain cancers. Neuro-Oncology Adv. (2024) 6 (1):vdae099. doi: 10.1093/noajnl/vdae099 PMC11259010 39036440

[100] Oberg JA Glade Bender JL Sulis ML Pendrick D Sireci AN Hsiao SJ. Implementation of next generation sequencing into pediatric hematology-oncology practice: moving beyond actionable alterations. Genome Med. (2016) 8:133. doi: 10.1186/s13073-016-0389-6 28007021 PMC5180407

[101] Newman S Nakitandwe J Kesserwan CA Azzato EM Wheeler DA Rusch M. Genomes for kids: the scope of pathogenic mutations in pediatric cancer revealed by comprehensive DNA and RNA sequencing. Cancer Discovery. (2021) 11:3008–27. doi: 10.1158/2159-8290.CD-20-1631 PMC8783930 34301788

[102] Parsons DW Roy A Yang Y Wang T Scollon S Bergstrom K. Diagnostic yield of clinical tumor and germline whole-exome sequencing for children with solid tumors. JAMA Oncol. (2016) 2:616–24. doi: 10.1001/jamaoncol.2015.5699 PMC5471125 26822237

