# Supplementary material for: Molecular characterization of gliomas and glioneuronal tumors amid Noonan syndrome: cancer predisposition examined
Source: Front Oncol. 2024 Sep 6;14:1453309. doi: 10.3389/fonc.2024.1453309 (PMC11412961; doi:10.3389/fonc.2024.1453309)
Supplement: Supplementary file 1 [file DataSheet1.docx]

Supplementary Material

**
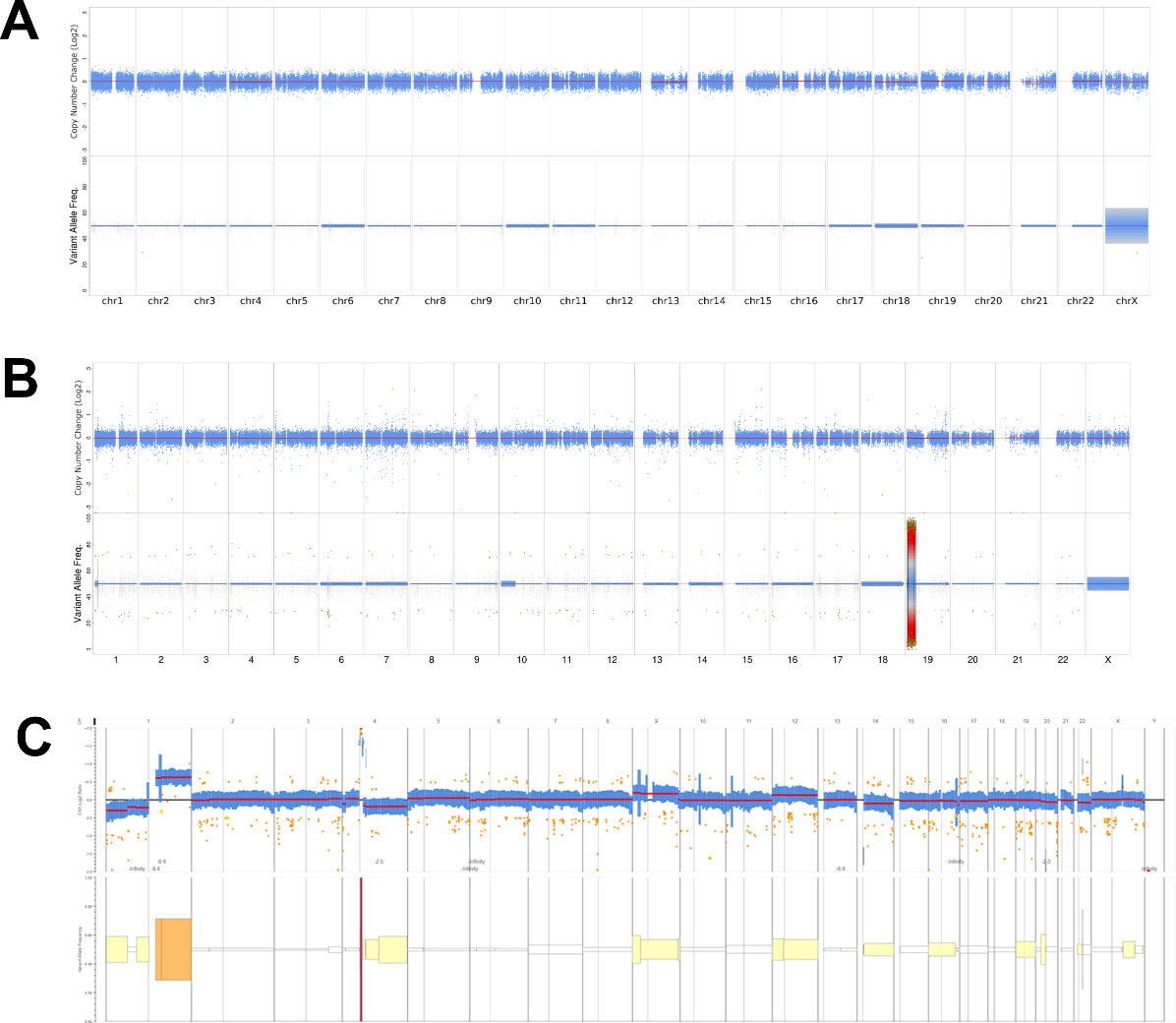
**

**Supplementary Figure 1.** **Tumor Copy Number Plots from the NCH Cohort.** Copy number variation (CNV) derived from exome sequencing data of the tumor relative to a panel of normals with copy number change (upper panel) measured in a Log2 scale. A Log2 ratio of zero is consistent with a two-copy state for autosomal chromosomes. Variant allele frequency deviating from 50% is depicted in the lower panel for each dataset. Individual 1 (A) with tumor displaying no apparent CNV or copy-neutral loss of heterozygosity (cnLOH). Individual 2 (B) with tumor displaying no apparent CNV, however cnLOH of the distal portion of chromosome 19p is detected. Individual 3 (C) with tumor displaying loss of chromosome 1q, gain of 1p, amplification of 4q12 (encompassing the *PDGFRA, KIT, KDR, CHIC2,* and *FIP1L1* loci), as well as other chromosome-level deviations.


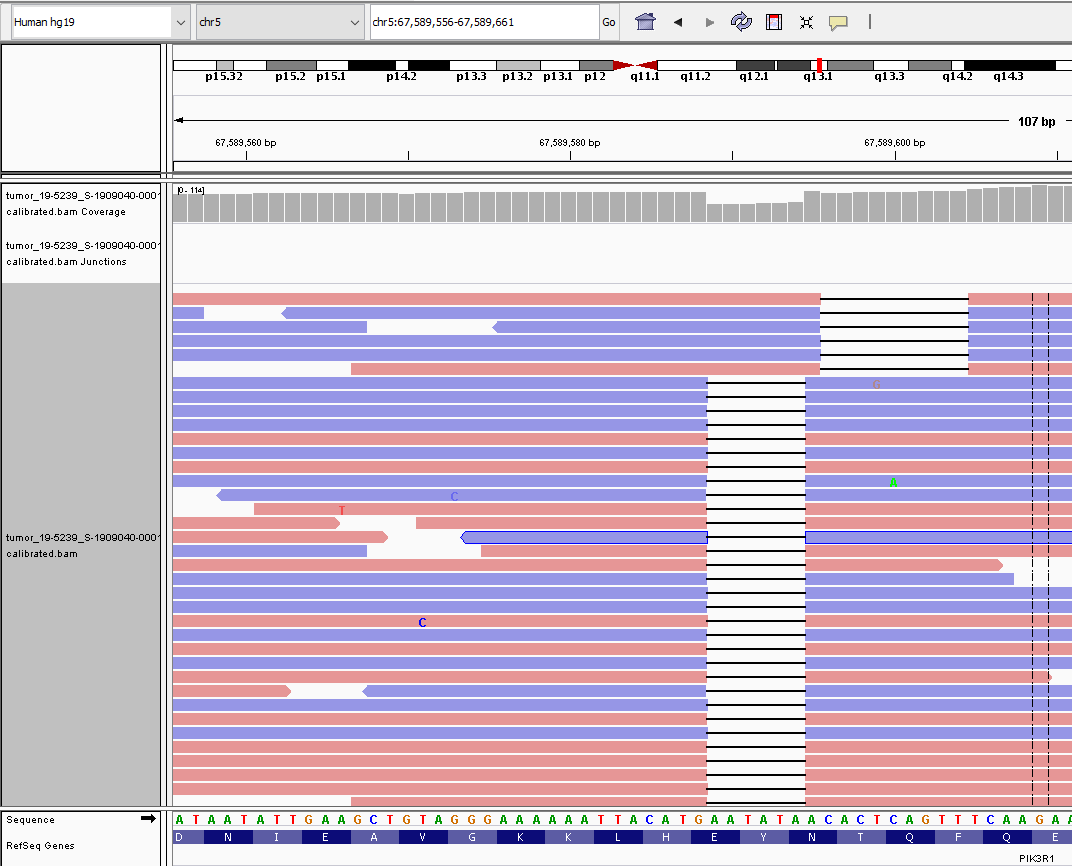


**Supplementary Figure 2.** ***PIK3R1* variants in Individual 2.** Sequence analysis identified variants at close genomic proximity in *PIK3R1* (*PIK3R1* p.Glu451_Asn453delinsAsp (34% VAF) and p.Thr454_Phe456del (7% VAF)). In each instance the variants within the gene occurred on differing sequencing reads in the aligned data, which is attributed to represent clonal heterogeneity within the tumor, as further supported by the differing VAFs. This region is enriched for insertion-deletion events associated with PI3K pathway activation and predicted to result in activation of the PI3K signaling pathway (1-3).


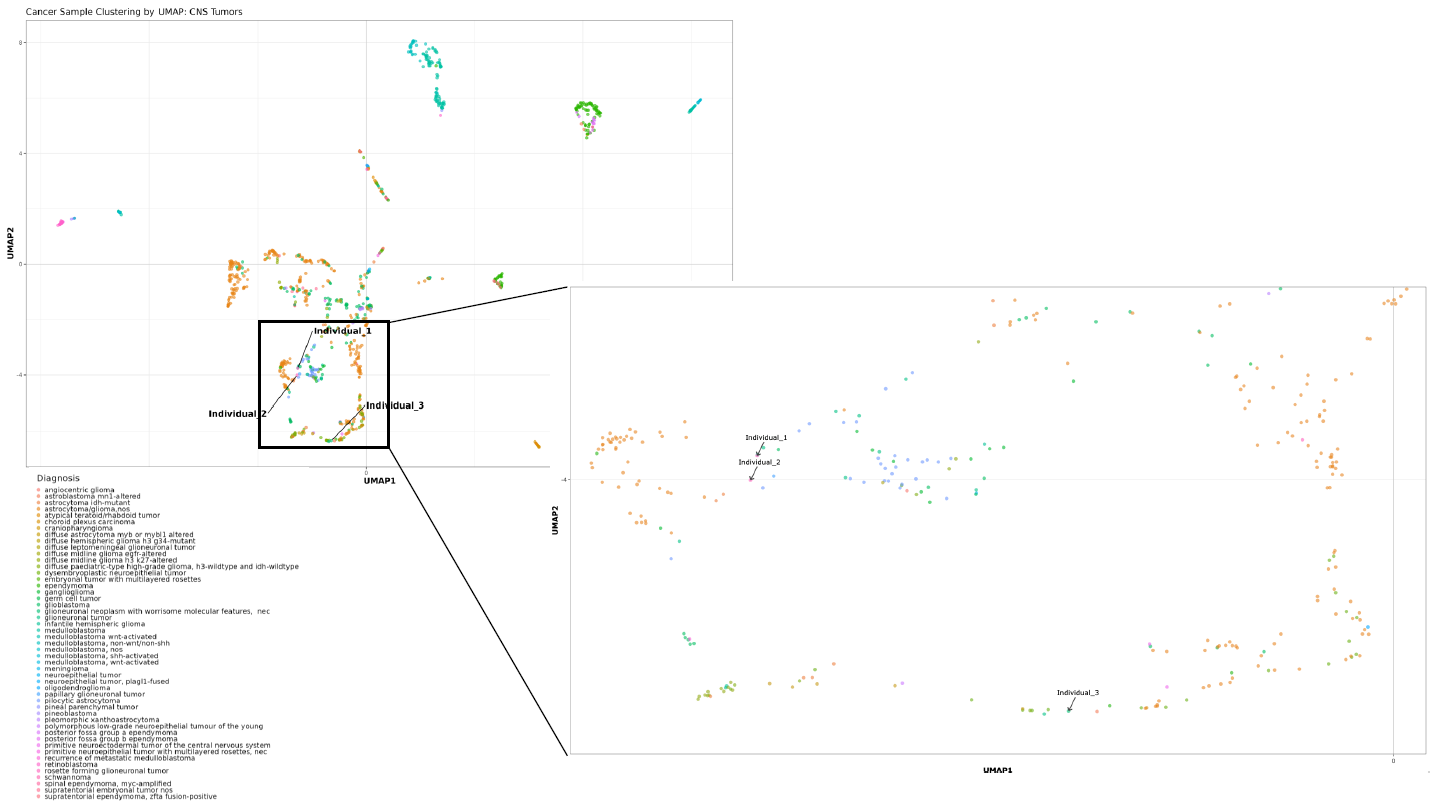


**Supplementary Figure 3. UMAP visualization of gene expression-based clustering of Individuals 1-3.** UMAP plot of complete dataset including pediatric CNS tumors from the Nationwide Children’s Hospital Institute of Genomic Medicine (n=235) and from the University of California Santa Cruz Genomics Institute’s Treehouse Childhood Cancer Initiative (n=791). The points are labeled by diagnosis. Individuals 1, 2, and 3 are marked by respective labels and arrows. The local clustering of the samples of interest is shown in greater detail on the right.


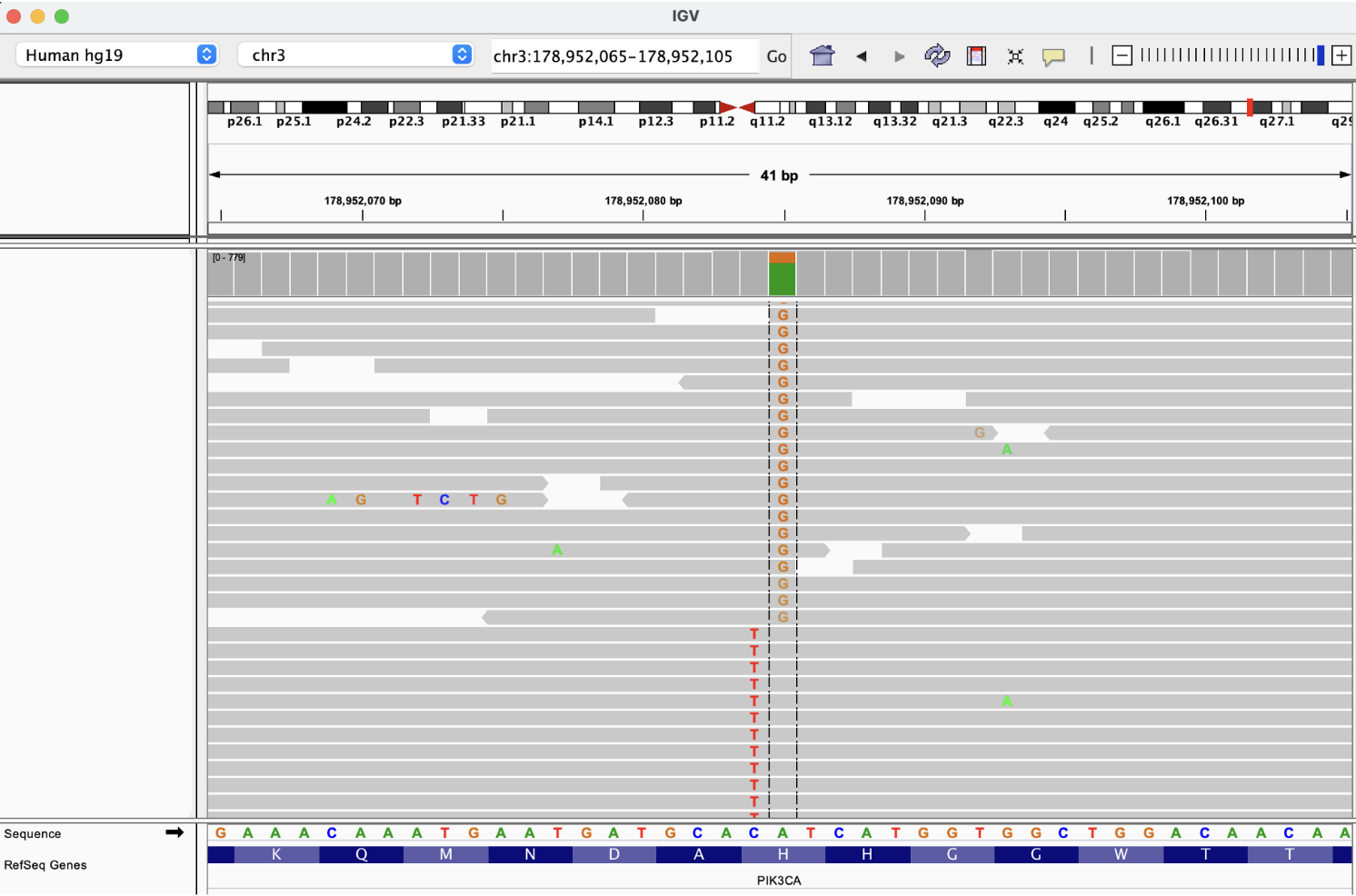


**Supplementary Figure 4. *PIK3CA* variants in Individual 4.** Sequence analysis identified variants at close genomic proximity in *PIK3CA* (p.His1047Tyr (5% VAF) and p.His1047Arg (25% VAF)). In each instance the variants within the gene occurred on differing sequencing reads in the aligned data, which is attributed to represent clonal heterogeneity within the tumor, as further supported by the differing VAFs. These alterations are predicted to result in activation of the PI3K signaling pathway. *PIK3CA* alterations *in cis* have demonstrated increased activation of the PI3K signaling pathway compared to single variants or variants *in trans* and resulted into hypersensitivity to PI3K inhibitors (4, 5). However, variants *in trans* demonstrated a response to targeted PI3K inhibition similar to a single alteration in *PIK3CA* (4).

**Supplementary Table 1: Sequencing Depth of Coverage**

|  | **Method** | **Comparator Germline Average Coverage** | **Tumor Average Coverage** |
| --- | --- | --- | --- |
| **Individual 1** | Paired exome sequencing | 261x (PB) | 255x (SP) |
| **Individual 2** | Paired exome sequencing | 221x (PB) | 214x (SP) |
| **Individual 3** | Paired exome sequencing | 301x (saliva) | 200x (SP) |
| **Individual 4** | UW-OncoPlex v7 | 697x (PB) | 819x (FFPE) |
| **Individual 5** | UW-OncoPlex v7 | 748x (PB) | 1180x (FFPE) |

PB=peripheral blood, SP=snap frozen, UW=University of Washington, FFPE=formalin-fixed, paraffin-embedded

**Supplementary Table 2: NCH Cohort Curated Gene List**

**Supplementary Table 3: University of Washington-OncoPlex version 7 Gene List**

**Supplementary Table 4: Clinical Features and Phenotypic Findings in Consideration of Noonan Syndrome Diagnosis**

|  | **Previously Established NS Diagnosis*** | **Short Stature** | **Dysmorphic Facial Features** | **Intellectual Disability/**  **Delay** | **Cardiac Abnormalities** | **Other Notable Findings** |
| --- | --- | --- | --- | --- | --- | --- |
| Individual 1 | Y | Y; Treated with growth hormone from age 5-14yrs | Downward sloping palpebral fissures, low set ears | Has an IEP to allow for extra time due to processing difficulty | VSD, aortic root dilation | Bilateral cryptorchidism, hydrocephalus, type I von Willebrand disease, adrenal insufficiency |
| Individual 2 | N | Y | Bilateral ptosis, triangular facies, coarse facial features | Y | Unknown | Scoliosis, bilateral ptosis, Chiari malformation, obstructive hydrocephalus, inguinal hernia |
| Individual 3 | N | Y; 3 %ile for height | Hypertelorism | Has an IEP for math and reading | ASD, pulmonary artery stenosis | Celiac disease |
| Individual 4 | N | Y | Hypertelorism | Y | Minor cardiac leaflet abnormalities | cryptorchidism, gastroesophageal reflux |
| Individual 5 | Y | Y | Triangular faces, slight hypertelorism, low set ears | N | N | Cryptorchidism, growth hormone deficiency |

*NS Diagnosis known prior to comprehensive molecular profiling in association with cancer diagnosis

Y= Yes; N= No; ASD= atrial septal defect; VSD= ventricular septal defect; IEP= Individualized Education Plan

**References**

1. Cottrell CE, Bender NR, Zimmermann MT, Heusel JW, Corliss M, Evenson MJ, et al. Somatic PIK3R1 variation as a cause of vascular malformations and overgrowth. Genetics in Medicine. 2021;23(10):1882-8.

2. Ye K, Wang J, Jayasinghe R, Lameijer E-W, McMichael JF, Ning J, et al. Systematic discovery of complex insertions and deletions in human cancers. Nature medicine. 2016;22(1):97-104.

3. Jaiswal BS, Janakiraman V, Kljavin NM, Chaudhuri S, Stern HM, Wang W, et al. Somatic mutations in p85alpha promote tumorigenesis through class IA PI3K activation. Cancer Cell. 2009;16(6):463-74.

4. Vasan N, Razavi P, Johnson JL, Shao H, Shah H, Antoine A, et al. Double PIK3CA mutations in cis increase oncogenicity and sensitivity to PI3Kalpha inhibitors. Science. 2019;366(6466):714-23.

5. Hutchinson KE, Chen JW, Savage HM, Stout TJ, Schimmoller F, Cortes J, et al. Multiple PIK3CA mutation clonality correlates with outcomes in taselisib + fulvestrant-treated ER+/HER2-, PIK3CA-mutated breast cancers. Genome Med. 2023;15(1):28.
